# A Man-Made ATP-Binding Protein Evolved Independent of Nature Causes Abnormal Growth in Bacterial Cells

**DOI:** 10.1371/journal.pone.0007385

**Published:** 2009-10-08

**Authors:** Joshua M. Stomel, James W. Wilson, Megan A. León, Phillip Stafford, John C. Chaput

**Affiliations:** 1 Center for BioOptical Nanotechnology, Arizona State University, Tempe, Arizona, United States of America; 2 Center for Infectious Disease and Vaccinology, Arizona State University, Tempe, Arizona, United States of America; 3 Center for Innovations in Medicine, The Biodesign Institute, Arizona State University, Tempe, Arizona, United States of America; 4 School of Life Sciences, Arizona State University, Tempe, Arizona, United States of America; 5 Department of Chemistry and Biochemistry, Arizona State University, Tempe, Arizona, United States of America; Center for Genomic Regulation, Spain

## Abstract

Recent advances in *de novo* protein evolution have made it possible to create synthetic proteins from unbiased libraries that fold into stable tertiary structures with predefined functions. However, it is not known whether such proteins will be functional when expressed inside living cells or how a host organism would respond to an encounter with a non-biological protein. Here, we examine the physiology and morphology of *Escherichia coli* cells engineered to express a synthetic ATP-binding protein evolved entirely from non-biological origins. We show that this man-made protein disrupts the normal energetic balance of the cell by altering the levels of intracellular ATP. This disruption cascades into a series of events that ultimately limit reproductive competency by inhibiting cell division. We now describe a detailed investigation into the synthetic biology of this man-made protein in a living bacterial organism, and the effect that this protein has on normal cell physiology.

## Introduction

The emerging field of synthetic biology is divided into two broad classes, both of which attempt to understand and harness basic underlying principles of living systems[1]. One uses engineering concepts to design and build artificial gene networks from component parts that exist in nature, but once assembled function in unnatural ways[2], [3], [4], [5]. Early efforts in this area have resulted in examples of engineered microorganisms, mainly *Escherichia coli* and *Saccharomyces cerevisiae*, with synthetic biology frameworks that function as toggle switches[6], self-assemble into oscillating networks[7], and establish predator-prey ecosystems[8]. A different area of synthetic biology relies on chemistry and chemical biology to develop unnatural chemical systems that emulate the emergent properties of life or function inside living cells[9], [10]. This area of synthetic biology is credited with establishing fundamental insights into the origins and biology of the cell[11], [12], [13], [14], [15], [16], as well as creating an alternative genetic system that is now used as a diagnostic tool to detect HIV and hepatitis in infected patients[17], [18].

One unexplored avenue within the chemical side of synthetic biology involves examining the physiology of man-made proteins inside living cells. While recombinant DNA technology has produced many examples where natural proteins have been engineered with properties that are desirable for biotechnology, such as improved stability or expanded substrate specificity, these structures all derive from sequences whose ancestors can be traced back to nature[19], [20], [21]. Recent progress in *de novo* protein design and *de novo* protein evolution has shown that it is now possible to create novel synthetic proteins using methods that no longer rely on natural protein scaffolds as starting points[22]. Examples of man-made proteins include a 4-helix bundle created by binary patterning[23], computational design of an α/β protein called Top7[24], and an *in vitro* evolved ATP-binding protein known as Family B[25]. While all three proteins adopt discrete structures, with Top7 and Family B collapsing into novel folds, only the *de novo* evolved ATP-binding protein exhibits a predefined function[25]. Our previous experience in the directed evolution and structure determination of the Family B protein led us to wonder how living cells might respond to an encounter with a man-made protein whose creation never involved heterologous expression in a host organism[26], [27], [28]. Would such an encounter reveal something new about biological pathways or help explain why certain protein folds are not observed in nature? One possibility is that natural selection may have biased the set of proteins found in nature to favor only those structures that are well suited to the cellular environment. This would suggest that proteins with non-cellular origins might have structures or functions that are incompatible with normal cellular biology, which of course would significantly limit their use in synthetic biology.

**Figure 1:**
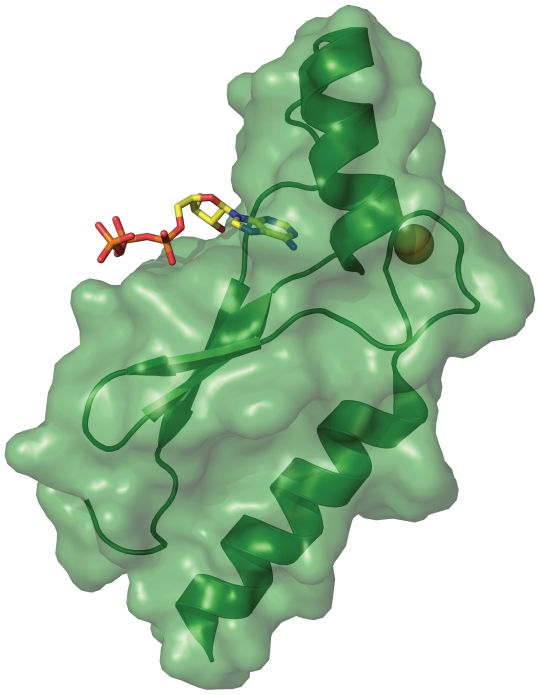
X-ray crystal structure of protein DX with translucent surface rendering. Protein DX adopts a novel zinc-nucleated α/β-fold with a topology not yet been observed in nature. The zinc and ATP ligands are colored according to atom type (PDB: 2P09).

We therefore designed a set of experiments that enabled us to monitor the physiology and morphology of *Escherichia coli* cells transformed with a plasmid containing the synthetic gene to a well characterized and highly evolved variant of the Family B protein known as protein DX. We previously evolved protein DX from its synthetic progenitor to bind ATP with high affinity and specificity, and solved the x-ray crystal structure of this protein to a resolution limit of 1.65 Å[28]. This man-made ATP-binding protein (Figure 1) adopts a novel zinc-nucleated α/β-fold with a unique topology. The ATP-binding motif differs considerably from traditional ATP-binding motifs found in nature[29], suggesting that there are many solutions to the problem of a how a protein can fold to bind ATP. While biochemical and structural characterization of protein DX required expression and purification from *E. coli* lysate, no systematic attempt has yet been made to characterize the interactions between this protein and any host organism. Given the importance of ATP as the main energy source of the cell and central metabolite and substrate in many enzymatic pathways, we reasoned that expression of protein DX in *E. coli* would elicit one or more biochemical responses due to the function of DX as a high affinity ATP-binding protein. The following study describes the first investigation into the synthetic biology of a non-biological protein in a living bacterium, and its consequences on cell physiology.

## Results

### Expression of a Man-Made Protein in Bacteria

**Figure 2:**
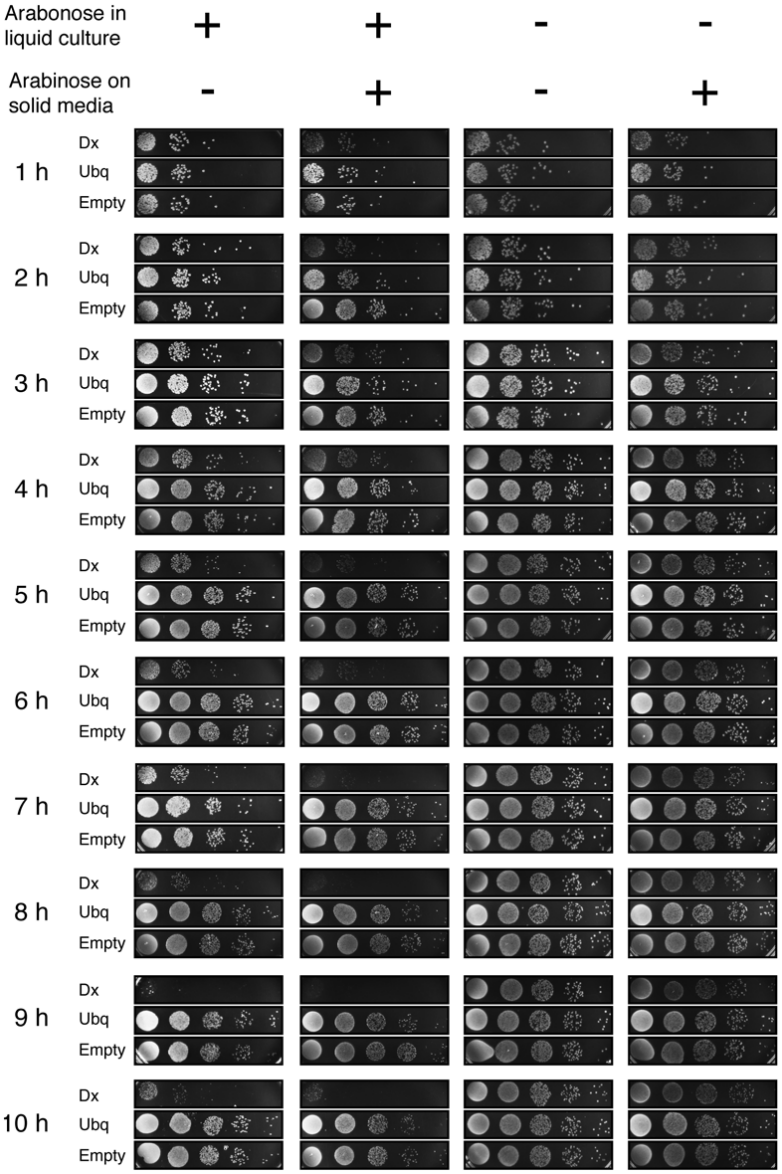
Expression characteristics of *E. coli* cells expressing protein DX. *E. coli* cells containing the DX, ubiquitin, or empty vector were induced in liquid media with arabinose and spotted in 10-fold dilutions onto solid agar plates that either contained (column 2) or were devoid (column 1) of the arabinose inducer. A series of control experiments were performed in parallel in which uninduced cells were plated onto solid media that contained (column 4) or were devoid (column 3) of arabinose.

To evaluate the effect of expressing a completely synthetic protein inside a living host organism, a pBAD18 vector containing the DX gene under the control of an arabinose-inducible promoter (pBAD18-DX) was transformed into the commercial *E. coli* strain Top10[30]. Control strains were constructed with an empty vector (pBAD18-E) and pBAD18 vector expressing human ubiquitin (pBAD18-UBQ). Ubiquitin was chosen based on its similar size to protein DX, well-folded nature, and absence of a native homologue in *E. coli*. We cultured all three strains in Luria-Bertani broth (LB) media containing arabinose inducer and monitored cell density and growth over the course of a 10-hour period. Cells were plated onto LB-agar plates in 10-fold serial dilutions, beginning with a 1000-fold dilution and colonies were inspected after overnight incubation at 37°C. We observed that the number of colony forming units (CFUs) present in the DX-producing strain increased during the first four hours of induction. However, after four hours of induced growth, the number of CFUs present in the DX-producing strain remained constant, while the number of CFUs observed in the control strains continued to increase with time (Figure 2). Close inspection of the CFUs formed by the pBAD18-DX strain indicated that at longer induction times the DX-producing colonies appeared smaller, darker, and more flat than colonies observed in either control strain (Figure S1). This effect manifests after only one hour when protein expression is maintained by plating the DX-producing strain onto solid media containing inducer. When the DX-producing strain is both cultured and plated in the absence of inducer the resulting CFUs appear indistinguishable from CFUs produced in the control stains, and only minor differences are observed when the DX-producing strain is cultured in liquid media without inducer but plated onto solid media containing the arabinose inducer.

### Metabolic Effect of DX Expression

**Figure 3:**
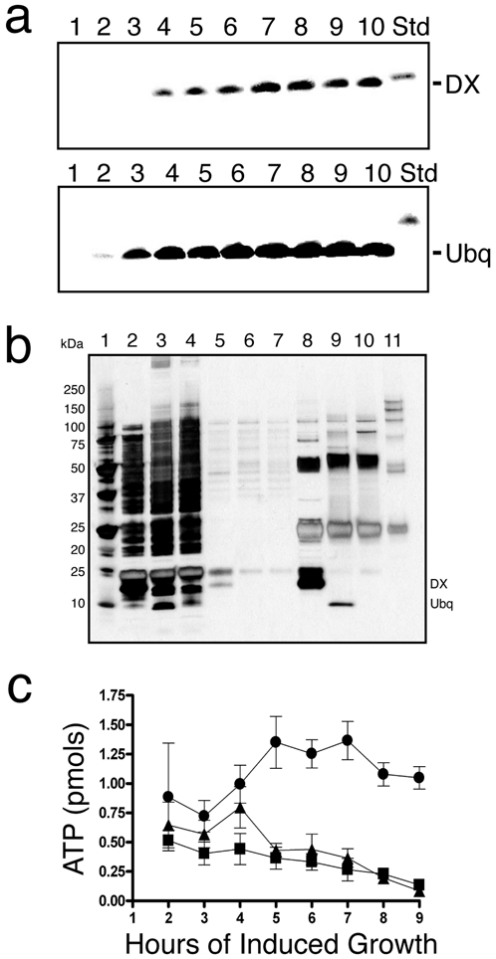
Biochemical properties of *E. coli* cells that express DX. (a) Western blot showing expression of DX and ubiquitin. The blots are normalized to a DX standard of 120 µg. Lanes 1–10 corresponds to 1–10 hours of induced growth. (b) Immunoprecipitation of protein DX. Lane 1, protein standards; lanes 2–4, crude lysate; lanes 5–7, wash fractions; lanes 8–10, immuno-precipitate. In each case, samples are given in order of DX, ubiquitin, and empty vector, respectively. Lane 11 is a bead only control. (c) Quantitative chemiluminescence analysis of ATP levels in cells expressing DX (•), ubiquitin (▴), and empty vector (▪).

Limited propagation of the DX-producing strain under post-induction conditions indicates that expression of the DX gene in *E. coli* leads to interference with the normal mechanism of cell growth and division. This type of response can occur when cells deplete their available nutrients by translating proteins at abnormally high levels [31]. To test this possibility, we monitored protein formation by western blot analysis using an anti-Flag antibody to detect a Flag epitope that was engineered into the amino-terminus of the DX and ubiquitin protein constructs. Strains expressing DX and ubiquitin were analyzed on an hourly basis following arabinose induction in liquid cultures. Inspection of the resulting gels (Figure 3a) revealed that protein DX is translated at lower levels and at later times than ubiquitin (4 versus 2 hours, respectively). Comparison of both time-course experiments to the DX standard in the control lane demonstrated that ubiquitin, which is present after two hours of induced growth, is produced at levels that are 3-4-fold higher than DX. This result indicates that the limited growth of the DX-producing strain is not due to broad nutrient depletion caused by protein over-expression, but rather a systemic effect in which protein DX interferes with one or more normal cellular processes.

We performed a standard immunoprecipitation assay to evaluate the possibility that protein DX was interfering with cell division by binding to one or more endogenous *E. coli* proteins. The DX, ubiquitin, and empty-vector strains were cultured for four hours with induction, lysed, and the cell contents were immobilized onto anti-Flag sepharose beads. The beads were thoroughly washed in buffer containing 0.1% nonidet-P40 and 1 mM DTT and the protein that remained bound to the resin was eluted with SDS and analyzed by denaturing gel electrophoresis. Silver-stained images (Figure 3b) of the resulting gels revealed the presence of two faint bands at 30 and 70 kDa, one intermediate band at 75 kDa, and one dark band at 25 kDa in the DX lane that were not present in the elution fractions taken from either control strain or the bead-only lane. Whether these proteins bind directly to DX or bind other *E. coli* proteins that bind to DX is not clear. It is also unclear whether these proteins have a role in the reduced-growth phenotype given that all but one are present at much lower concentrations than protein DX. The observation that protein DX does not bind to many proteins inside the cell is interesting, and suggests that protein DX is not any stickier than a typical protein found in nature. This result is surprising, given that protein DX was created by a purely cell-free method with no direct selective pressure to function inside a cell.

Because ATP is the primary energy source of the cell and an important substrate in many biological pathways, we reasoned that the function of protein DX as an ATP-binding protein might interfere with cell division by altering the level of free ATP inside the cell. To examine this possibility, we monitored the intracellular levels of ATP over the course of a 10-hour period. We cultured all three strains in LB media containing inducer, and measured the amount of intracellular ATP on an hourly basis using a highly sensitive bioluminescent assay. Cell samples were normalized to total cell protein and a standard linear calibration was used to determine the absolute quantity of ATP in each sample. In this assay, the ATP levels in the DX strain remained within one standard deviation of the control strains during the first three hours of induced growth (Figure 3c). After three hours, ATP levels in the DX-producing strain begin to rise, while ATP levels in the control strains fall. This trend continues until a point at which a ∼10-fold difference is evident between the DX and control strains. The fact that intracellular levels of ATP begin to change at the same time that protein DX is first detected inside the cell, and that both occur contemporaneously with the transition of the DX-producing strain from normal to reduced cell growth, is consistent with the hypothesis that the function of DX as an ATP-binding protein is responsible for the reduced growth phenotype.

### Reversibility of the DX Phenotype

**Figure 4:**
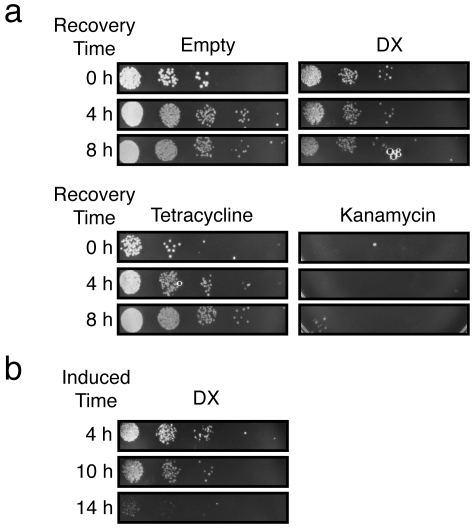
Reversibility of DX growth suppression. (a) *E. coli* cells cultured in the presence of inducer for 10 hours were allowed to recover in the absence of inducer for a variable amount of time (0–8 hours). Cells expressing protein DX were compared to control cells containing the empty vector exposed to a bacteriostat (tetracycline), bactericide (kanamycin), or no additional antibiotic. (b) *E. coli* cells were cultured in the presence of inducer for varying amounts of time (4–14 hours) and allowed to recover for 4 hours in LB media free of inducer.

The observation that our synthetic protein was having a dramatic effect on cell proliferation led us to wonder whether DX functioned as a bactericidal or bacteriostatic agent. To examine this question in greater detail, we cultured the DX-producing strain for 10 hours under induction, removed the inducer and allowed the cells to continue growing for an additional eight hours. Cells taken during the recovery period were plated onto LB-agar plates in 10-fold serial dilutions. The resulting plates (Figure 4a) were compared to control cells in which the empty vector was separately cultured in the presence of the bacteriostatic agent tetracycline and the bactericidal agent kanamycin or in the absence of either antibiotic. We observed that under our expression regime, protein DX appears to function as a bacteriostatic agent since the DX strain displayed a phenotype that is similar to the tetracycline control. To confirm this observation, we used a time course experiment to monitor CFU formation during eight hours of expression and eight hours of recovery. We then performed a two-way ANOVA analysis with respect to time and cell culture type to determine whether the differences observed were statistically significant (Figure S2, Table S1). We found that there were significantly fewer cells in the DX-expressing culture than either the empty or ubiquitin control cultures (p-value<0.001) after three hours of recovery in the absence of arabinose. During the expression period, the number of cells observed in the DX culture did not differ significantly from the tetracycline control even though the tetracycline culture had a lower number of CFUs at each time point. We noticed that cells containing the empty vector appear to exert a slight increase in abundance after removal of the arabinose inducer. This effect is presumably due to a small inhibitory effect of arabinose on the growth rate of *E. coli*. The observation that the DX-expressing culture behaves similar to the tetracycline treated culture supports our hypothesis that the DX protein functions as a bacteriostatic agent.

However, unlike cells grown in the presence of tetracycline, which propagate normally when re-suspended in antibiotic-free media, the DX-producing strain exhibits a delay in its recovery response when re-suspended in LB media devoid of inducer. Despite the fact that colony maturity was quickly restored, the number of CFUs observed in the DX-producing strain did not increase in abundance during the recovery period. To determine if this lag in cell division is related to abnormalities observed in colony appearance, we repeated this experiment by varying the induction time while maintaining a constant recovery period of four hours in LB free of inducer. Cells induced for 4 and 10 hours resulted in colonies that are indistinguishable from the tetracycline control. Cells induced for 14 hours produced colonies (Figure 4b) that are similar in appearance to colonies observed when protein expression is maintained by plating cells onto solid media containing inducer (Figure S2). This result is consistent with our earlier observation that colony abnormality is more severe when protein expression is maintained for extended periods of time, and suggests that longer expression times leads to the accumulation of large numbers of unhealthy cells.

### Light Microscopy Studies

**Figure 5:**
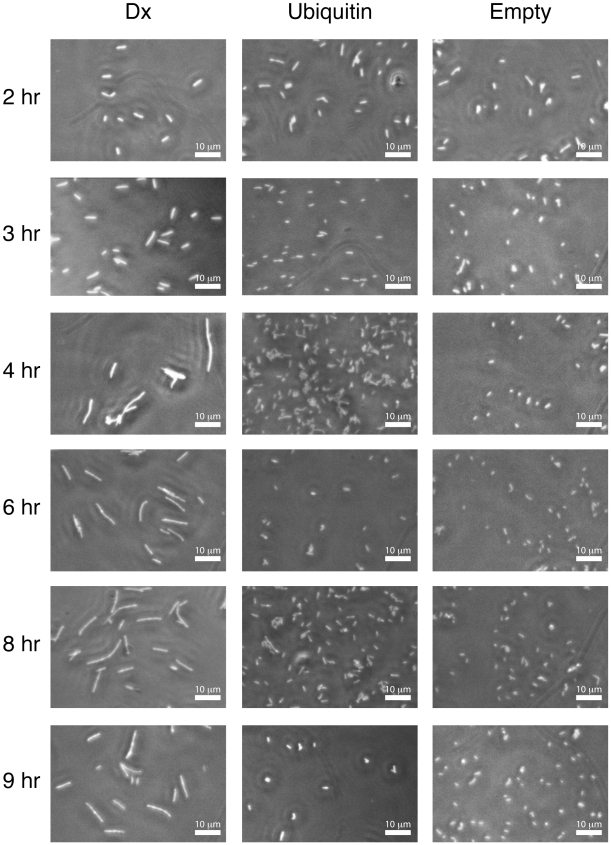
Analyzing the effect of DX expression on cell morphology. Phase-contrast micrograph images taken between 2 and 9 hours post-induction show the filamentous phenotype in cells expressing protein DX. The unbiquitin and empty control strains appear normal at all times of induced growth. Images were acquired at 40× magnification.

We used phase contrast microscopy to examine the morphology of *E. coli* cells that were actively expressing the synthetic DX gene. We were particularly interested in determining whether DX had an effect on cell growth, as some bacteriostatic agents will inhibit growth and division of a cell, while others will inhibit only division but allow cell growth to continue[31]. Bacteriostatic agents that only inhibit cell division result in a filamentous phenotype that is easily observed by light microscopy. In a nine-hour time course experiment (Figure 5), cells taken within the first three hours of induction appeared indistinguishable from the control cells. After three hours, cells taken from the DX strain began to filament, while the control strains remain normal. Close inspection of individual micrographs indicated that the length of filamentation increases with induction time. To quantify this progression, we compiled a statistical sampling of cells and measured the length of these cells after each hour of induction. The median length of cells expressing DX increased from 3.2 to 6.1 µm when cells were induced for two and six hours, respectively. After six hours, the rate of cell elongation stabilizes to a point at which the population of cells have a median length of ∼6.5 µm. No difference was observed between un-induced cells that contain the pBAD18-DX plasmid and the control cells, and both of these cells remained within one standard deviation of their initial value. This result demonstrates that the DX-strain has two cellular phenotypes—one that leads to cell filamentation and one that causes the cessation of further cell elongation.

**Figure 6:**
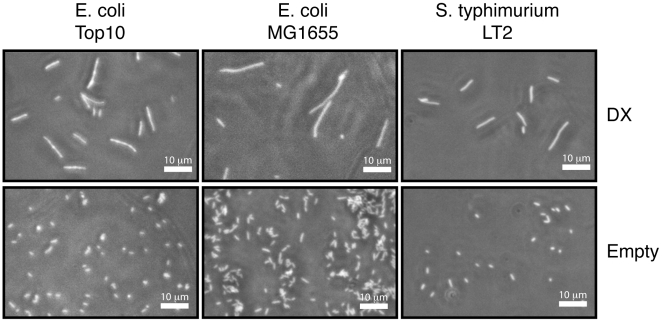
Species and strain specificity of DX filamentous phenotype. Phase-contrast micrograph images of *E. coli* TOP10, *E. coli* K12 MG1655, and *S. typhimurium* LT-2 taken at 9 hours post-induction of the DX protein. Control cells of each strain containing the empty vector are included to provide a basis of comparison. Images were acquired at 40× magnification.

Encouraged by the observation that protein DX functions as a bacteriostatic agent, we transformed the pBAD18-DX and pBAD18-E plasmids into the MG1655 strain of *E. coli* K-12 and *S. typhimurium* strain LT-2 to determine if the filamentous phenotype could be elicited in other types of bacteria. MG1655 represents a less engineered form of *E. coli* relative to the Top10 strain, which is heavily modified for laboratory use. Phase contrast microscopy (Figure 6) reveals that expression of DX in these two strains results in the same filamentous phenotype observed in the TOP10 strain. This demonstrates that DX expression disrupts bacterial growth in multiple bacterial strains, which suggests that DX is targeting a conserved bacterial pathway.

### Transmission Electron Microscopy Studies

**Figure 7:**
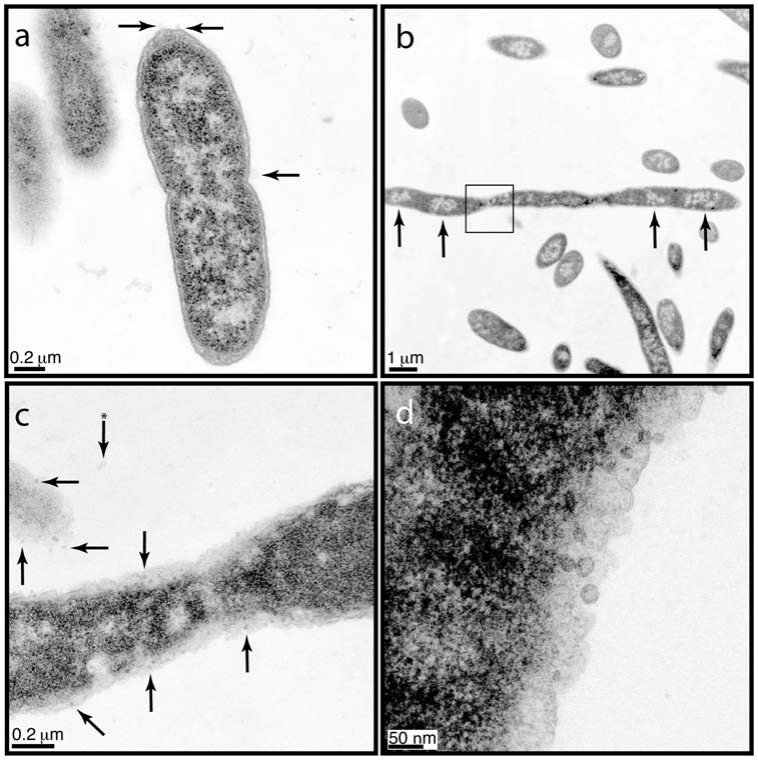
Electron micrographs of thin sections through embedded *E. coli* expressing DX. The cells were cultured for 3 hours in liquid media in the absence (a) or presence (b–d) of inducer. (a) An uninduced cell displaying a forming septum and three membranous spheres marked by arrows. (b) An example of a filamentous cell expressing DX. Areas marked by arrows indicate regions of chromosomal segregation. (c) Increased magnification of the boxed area in (b). A selection of membranous structures associated with the surface of the cell is marked with arrows. The arrow marked with a * indicates structures which have become detached from the cell. Images examined both above and below the plan reveal that this portion of the cell is not forming a division septum. (d) High magnification image of several spherical structures.

We used transmission electron microscopy (TEM) to examine the filamentous phenotype at higher resolution. We analyzed the DX-producing strain in *E. coli* Top 10 cells after three, four, and five hours of induced growth. We focused our analysis on data obtained after three hours as these micrographs produced the highest quality images. Micrographs taken of uninduced cells show *E. coli* cells at various stages of cell division. All of the uninduced cells appear healthy and range in length from 1–2 µm with the longest actively dividing cell having a length of 2.2 µm. Illustrated in figure 7a is a typical example of a healthy bacteria cell undergoing division. The micrograph shows the separation of chromosomes into dividing cells and the initial formation of a division septum at the cell center. The cell wall and cytoplasmic membrane are clearly visible, as are ribosomes, which stain as dark spots scattered throughout the cytoplasm. In contrast, micrographs taken after induction (Figure 7b) show cells that have grown into long filaments, some of which exceed 12 µm in length. Filamentous cells sectioned along their longitudinal axis contain multiple well-defined nucleoids that segregate evenly along the length of the cell with ribosomes scattered throughout the cytoplasm. None of the induced cells have a septum, indicating that these cells are not actively dividing. However, these cells do contain large numbers of vesicles (Figure 7c–d) in their outer membrane, which could suggest that the host organism is trying to remove protein DX from the cell. In one case we observed 21 vesicles along a short 1.7 µm section of membrane. Only three such structures were observed in a typical control cell of similar length. These images demonstrate that the filamentous phenotype is not caused by failure of chromosomes to divide or segregate [31], but rather some other mechanism that interferes with cell division.

### Differential Gene Expression by Microarray Analysis

We examined the effects of DX expression on the global regulatory pathways of *E. coli* using a time-dependent microarray analysis assay[32]. We isolated cellular RNA at half-hour intervals over a 4-hour period starting with induction. Cellular RNA was reverse transcribed into cDNA with Alexa-647 and Alexa-555 fluorescent dyes used to label the cDNA from induced and uninduced cells, respectively. Pairs of differentially labeled cDNA samples from each time point were hybridized to a custom Agilent *E. coli* expression array on which eight sets of the 4,169 *E. coli* K-12 open reading frames (ORFs) were printed. The expression data was extracted using Genepix, loaded into GeneSpring 7.2 and filtered according to flags and reproducibility. We first selected genes that change under a steady-state model of increasing or decreasing expression over time. We then used regression analysis to match a theoretical pattern of expression, which increases or decreases smoothly over the 4-hour period. From this analysis, we were able to identify 195 genes whose expression matched the rising pattern (Table S2) and 184 genes whose expression matched the declining pattern (Table S3). Of the 195 genes that rose over time, 44 represent ORFs of unknown function. The remaining genes, when analyzed using the Database for Annotation, Visualization, and Integrated Discovery (DAVID)[33], fell into four clusters of categories: oxidoreductases, taurine and sulfur metabolism associated genes, membrane proteins, and flagellar and chemotactic genes. Each of these clusters contained multiple gene ontology classifications that were over represented when compared with a random list of *E. coli* genes of similar size. We considered Gene categories to be significantly over-represented if DAVID returned a P-value of 0.1 or less.

Based on these initial findings, we decided to perform a more extensive analysis of genes related to the over-represented ontological categories. A genome-wide analysis of all genes annotated with the ontology term “taxis” reveals that many of these genes show mild time-dependent up-regulation in cells that express the DX gene (Table S4). This includes significant up-regulation of *flhC/D*, which acts as a sigma factor for class II flagellar genes[34]. Genome-wide analysis of all genes annotated with the gene ontology term “sulfur metabolism” reveals that while many show a mild time-dependent up-regulation, several, especially the *sufABCDES* genes, show a strong time dependent down-regulation (Table S5). Oxidoreductases and membrane proteins were not analyzed at a genome wide level due to the number and variety of genes involved in these pathways, which will require an independent analysis.

Of the 184 genes that decline in expression over time, 38 were ORFs of unknown function. When analyzed with DAVID, the remaining genes segregate into six broadly defined categories: signal proteins, phosphoric ester hydrolyses, amino acid and nitrogen compound biosynthetic proteins, disaccharide metabolism proteins and glycosidases, lipoprotein and lipid binding proteins, and pyrimidine nucleotide biosynthetic proteins. Since *rpoS* is known to be an important transcriptional regulator[35], and linear regression analysis indicated that it matched a trend of gradual reduction in expression over time, we examined the expression of genes that this protein regulates. We found that the majority of genes identified as being induced by *rpoS* were repressed in cells expressing the DX gene (Table S6). This is interesting because *rpoS* is involved in regulating the transition from exponential to stationary growth, which is delayed or inhibited in cultures that express the DX gene. Of course, *rpoS* is also involved in the stress response caused by many other stimuli, so it is unclear if the drop in *rpoS* expression is related to expression of the DX gene.

Based on the DX-induced filamentous phenotype, we examined constituents of the SOS pathway and purine biosynthesis pathway, as well as a selection of genes involved in cell division and ATP synthesis. Genes in the SOS pathway, which is the primary means by which *E. coli* enters into filamentous growth[31], show no consistent up-regulation over time (Table S7). In fact, *sulA*, the central cell division suppression protein of the SOS pathway, is reduced in expression by more than two-fold over the course of 4 hours, while *lon*, which deactivates *sulA*, increases in expression during that same period of time. This is the opposite expression pattern that one would expect for an SOS response[31]. Examination of other genes involved in cell division shows no suppression of the *fts* cell division proteins or the *minCDE* proteins involved in proper placement of the *ftsZ* ring complex (Table S8)[31], however, *zapA*, which promotes polymerization of the FtsZ ring, is down-regulated nearly four-fold. To determine if the rise in cellular ATP levels observed in our ATP quantification assay (Figure 3c) could result from greater activity in ATP biosynthesis, we examined the expression levels of purine biosynthesis and ATP synthase genes (Table S9). The majority of genes involved in purine biosynthesis were either down regulated or unaffected following expression of DX; and *purR*, the repressor of purine biosynthesis, was up-regulated. In contrast, elements of the purine salvage pathway involved in converting adenine to ATP (*apt, adk*, and *ndk*), and in converting guanosine derivatives into AMP (*guaC*) were up-regulated. Additionally, six out of the nine ATP synthase genes (*atpABEFHI*) increased in expression. One interpretation of this result is that the cell is responding to a perceived lack of ATP, due to sequestration by DX, which causes an increase in ATP salvage pathways rather than *de novo* biosynthesis pathways.

## Discussion

The central focus of the current study was to investigate the synthetic biology of an artificial protein inside the environment of a bacterial host organism. We were motivated by the following question: how would a natural system respond to an encounter with an unnatural protein obtained from non-biological origins? Would the expression of a completely man-made protein in a living host organism reveal something new about biological pathways or help explain why certain protein folds are not observed in nature? Because our current understanding of even the most basic of living systems remains limited, investigations such as this have the ability to provide new insights into biological systems. Information gained from these studies could, in principle, be used to create novel synthetic systems that function in unnatural ways or possibly discover alternative avenues for therapeutic intervention. In addition, these types of studies also provide a unique opportunity to evaluate the properties of man-made proteins in living systems, as tailor-made proteins have the potential to endow living systems with synthetic functions that are not found in nature.

During the course of our study, we discovered that *E. coli* cells transformed with the DX plasmid experience reduced reproductive competency after three hours of induced growth. This change in cell growth is contemporaneous with the first detectable occurrence of DX in the cell, and is associated with an observed rise in intracellular ATP, the transition to a filamentous state, an increase in membrane vesicles, and a general slowing of cell metabolism. The filamentous phenotype was observed in multiple bacterial strains, which suggests that DX is targeting a conserved biological pathway. Microarray analysis showed a downward trend in pathways that involve the synthesis of important cell building blocks, such as amino acids, nucleotides, and disaccharides. These observations are consistent with the interpretation that cells expressing the DX gene are attempting to conserve ATP through a defense mechanism that lowers cellular activity along certain ATP-dependent pathways. While filamentation could be a response to cell stress[36], the absence of an SOS response in the microarray data leads us to suspect that its cause is more biochemical than genetic. In addition, down regulation of *zapA* could help facilitate cell filamentation, since ZapA is known to counteract FtsZ inhibition by MinC[37]. Within this same timeframe, a general upward trend was observed in genes involved in cell motility, sulfur metabolism, and ATP synthesis by salvage pathways. These genes are consistent with the ability of *E. coli* to escape harmful environments and activate secondary pathways for ATP synthesis.

It is interesting that many of the genes found to be over-represented in our study were also identified in another study in which artificial gene networks using natural transcription factors in novel regulatory networks were constructed in *E. coli* Top10 cells[38]. In that case, it was discovered that artificial gene networks containing the *flhD* or *rpoS* promoter regions conveyed a selective advantage on *E. coli* growth and survival. The authors speculated that this advantage was due to interference between the artificial regulatory network and native networks that use the same component parts. It is intriguing that these same two regulatory elements were found to be over-represented in *E. coli* Top10 cells expressing the DX gene. Since disruption of the *flhD* gene has been shown to increase cell division[39], it may not be surprising that over-expression of *flhD* in DX expressing cells is associated with a decrease in cell division. While the authors found that *E. coli* is relatively tolerant to the introduction of new regulatory gene networks, albeit from natural sources, our study leads to the opposite conclusion, at least with respect to the use of synthetic proteins from non-natural origins. However, it remains to be seen whether protein DX is representative of a typical non-biological protein, or whether the difference between naturally derived and unnatural components plays an important role in the tolerance of *E. coli* cells to artificial parts and networks.

Based on the data collected, we suggest that the function of DX as an ATP-binding protein is responsible for disrupting the energetic balance within the cell. Several lines of evidence indicate that DX remains folded when expressed in *E. coli*. These include the fact that protein DX was previously crystallized from the soluble fraction of *E. coli* lysate[28], the absence of inclusion bodies in the electron micrographs, and the observation that induced growth leads to an ATP-related phenotype. This hypothesis is further supported by the observation that our gene expression data were able to identify extensive genetic changes relative to an earlier microarray study in which cell filamentation was induced by an external chemical agent[40]. We favor a model in which protein DX causes the cell to behave as if it were nutrient depleted and react by lowering general cell metabolism and increasing secondary ATP synthesis pathways. We speculate that the change in intracellular levels of ATP disrupts cell division by interfering with ATP-dependent proteins like MinD and FtsA, which are responsible for positioning the division septum at the cell equator[41], [42]. We suspect that this defect in cell division leads to a filamentous phenotype in which the cell is no longer able to propagate normally, and this phenotype persists until DX induction is halted and energetic balance is restored to the cell. Similar inhibitory effects have been observed in *E. coli* cells expressing non-coding intergenic regions of their genome[43]. However, it is unclear whether the peptides encoded by these regions form stably folded structures with discrete functions. It is interesting that the degree of filamentation observed in bacteria that are actively expressing the DX gene is less extensive than the extent of filamentation observed in other bacterial systems [44]. This observation, in combination with the fact that DX-expressing cultures continue to form colonies when plated onto solid media containing inducer suggests a model where division either slows down dramatically or frequently produces nonviable progeny. It is possible that increased cell stress coupled with altered ATP levels might be causing the cell to relieve this stress by exporting the DX protein from the cell[45]. This hypothesis is supported by the large numbers of outer membrane vesicles observed in the electron micrographs of filamentous cells. Future studies that examine cell physiology and morphology during the recovery period will help clarify the final state of the cell.

One unanticipated result to come from this study was the observation that DX functions as a bacteriostatic agent with an efficacy that is qualitatively similar to commercial tetracycline. This was an interesting result because its potential mode of action differs considerably from traditional antibiotic agents. Currently, almost all drugs used to treat bacterial infections target one of four general mechanisms: i) cell wall synthesis; ii) protein synthesis; iii) nucleic acid synthesis; and iv) metabolite synthesis[46]. Of the antibiotics that inhibit metabolite synthesis, most target folic acid synthesis, which is essential for the synthesis and maintenance of new cell walls[46]. Only a few examples exist where ATP has been implicated as a possible target for antibacterial activity. Nisin, a polycyclic peptide antibacterial agent commonly used as a food preservative, is one example of an antibiotic that depletes intracellular ATP levels[47]. However, unlike DX, which binds ATP directly, this agent causes cell death by disrupting the ion potential and pH gradient across the cell membrane[47]. Although it is still premature to determine whether ATP is a viable target for next-generation antibacterial agents, the demand for such drugs warrants further study of synthetic proteins like DX in cellular systems[48].

Unlike all previous examples of protein evolution, which start with a protein found in nature and optimize for such properties as improved folding stability or alternative function, our protein originated from an unconstrained pool of synthetic random sequences. When we began our study is was not clear that a protein whose entire evolutionary history involved a series of *in vitro* selection steps in a test tube would function inside a living cell. The fact that protein DX elicits an ATP-dependent phenotype in *E. coli*, warrants an explanation in terms of protein structure and the evolutionary forces that give rise to stably folded proteins. One hypothesis that is consistent with our observations and the general history of protein evolution is that protein structures are highly plastic, and thus any selective pressure, whether man-made or natural, that drives a protein toward improved stability has the potential to give rise to a native structure. One implication of this hypothesis is that cellular origin is not the only determinant of whether a protein will remain functional in a host organism. Testing this hypothesis will require examining the structural and functional properties of many additional examples of proteins that have been evolved in the absence of biological constraints.

In summary, the current study provides the first in-depth analysis of a non-biological protein in a living host organism. We found that a synthetic ATP-binding protein from non-natural origins functions inside living cell by disrupting the normal energetic balance within the cell. This disruption cascades into a series of events that limit reproductive competency by inhibiting cell division. This discovery provides a paradigm where synthetic proteins could be used to as novel therapeutics, including next generation antibiotics, and provides new opportunities for probing many basic and applied questions in cellular biology.

## Materials and Methods

### Bacterial Strains, Media and Growth Conditions

All experiments performed in this study involved *E. coli* TOP10 cells (Invitrogen) unless otherwise stated. Liquid cultures were grown at 37°C for 14–16 h in Luria-Bertani (LB) media containing ampicillin (US Biological, 200 µg/ml), and protein expression was induced by diluting the culture 100-fold into fresh LB containing arabinose (1 mg/ml).

### Construction of Plasmids

The DX and ubiquitin genes were amplified by the polymerase chain reaction (PCR) from a pmal plasmid [28]. The forward DNA primer contained the sequence information necessary to add an *E. coli* optimized Shine-Dalgarno sequence upstream of the open reading frame and modify the N-terminus of each protein to include a Flag protein affinity tag (MDYKDDDDK). In addition, the PCR primers also contained *Eco*RI and *Hin*dIII restriction enzyme sites, which were used to clone both genes into the pBAD18 plasmid[30]. Codon usage for the DX and ubiquitin genes was optimized for *E. coli* expression and proper insertion of both genes was verified by DNA sequencing.

### Cell Growth, Expression and Metabolism Studies

Liquid cell cultures containing the DX, ubiquitin, and empty pBAD18 vector were induced with arabinose. Over the course of 10 hours, four 1 ml aliquots and one 0.5 ml aliquot were removed at each one-hour interval. The 0.5 ml aliquot was used to measure cell density at 600 nm using an Eppendorf BioPhotometer. Each of the 1 ml aliquots were pelleted by centrifugation, frozen at −80°C, thawed on ice, and used in the following manner: the first aliquot was re-suspended in sterile water, lysed by sonication and total cell protein was determined by taking the average of three Bradford measurements; the second aliquot was re-suspended in 1x SDS containing loading buffer and used in a western blot assay, the third and fourth aliquots were re-suspended in pH 7.4 PBS and used to measure intracellular ATP levels. In addition, a 1 µl sample from each cell type was also removed after each hour of expression, diluted 1000-fold into sterile water, and spotted as a series of four 10-fold dilutions onto LB-agar plates that either contained or were devoid of arabinose (1 mg/ml). The cultures were grown on solid media for 18 hours at 37°C and imaged using a Bio Rad Gel Doc XR+ system. This experiment was performed in duplicate using liquid cultures that were grown in the presence or absence of arabinose.

### Western Blotting

Cell samples obtained in the cell growth study were re-suspended in 1x SDS containing loading buffer (Invitrogen). Sample volumes were normalized based on the total protein content determined by the Bradford assay. These samples were boiled for 5 min and analyzed by SDS polyacrylamide gel electrophoresis (SDS-PAGE). The resulting gel was transferred to polyvinylidene difluoride (PVDF) or nitrocellulose membranes using the Invitrogen iBlot system and FLAG-tagged recombinant proteins were visualized with horseradish peroxidase (HRP) using an anti-FLAG antibody (Immunology Consultants Laboratory) and with a chemiluminescent substrate (SuperSignal, Thermo Scientific).

### Intracellular ATP Assay

Cell samples obtained in the cell growth study were analyzed using the BacTiter-Glo microbial cell viability assay kit (Promega) and bioluminescence was detected using a Promega Glomax luminometer. Cell samples were normalized to total cell protein as determined by Bradford assay such that the total amount of protein in each sample was equivalent. A linear calibration was performed using known quantities of ATP (Sigma) to determine the absolute quantity of ATP in each sample, and all values represent the average of three trials.

### Immunoprecipitation Assay

*E. coli* TOP10 cells transformed with pBAD18 containing either DX, ubiquitin, or the empty vector were induced for 4 h at 37°C . Cells were harvested and lysed in buffer containing 1 mg/mL lysozyme, 50 mM Tris HCl (pH 7.5), 100 mM NaCl, 10% glycerol, 1 mM dithiothreitol (DTT), 0.1% Nonidet-P40, 1 mM phenylmethanesulphonylfluoride (PMSF), and protease inhibitor cocktail (Roche). Lysate was clarified by centrifugation at 21,130 *Xg* for 45 min at 4°C and filtered through a 0.22 µm sterile syringe filter unit. DX was immobilized by adding 1 mg of crude protein (determined by Bradford assay) to ANTI-FLAG® M2-Agarose Affinity Gel (Sigma) in binding buffer (similar to the lysis buffer, but without lysozyme and glycerol and supplemented with 15 mM EDTA). Protein and affinity gel were incubated for 2 h at 4°C, washed three times in wash buffer (binding buffer without protease inhibiter cocktail). Samples were analyzed by SDS-PAGE gel (NuPAGE, Invitrogen) and imaged using SilverXpress silver staining kit (Invitrogen).

### Toxicity Analysis

Cells containing the DX plasmid were cultured for 10 hours under induction. These samples were centrifuged at 3000 Xg at 4°C for 5 min, washed twice with fresh LB, and re-suspended in the same volume of LB devoid of the arabinose inducer. Over the course of an 8 hour experiment, cell aliquots (1 µl) were diluted 1000-fold into sterile water and spotted as a series of four further 10-fold dilutions onto LB-agar plates. A series of control cells containing the empty pBAD18 vector were cultured in an identical manner with the exception that these cells either contained kanamycin (US Biological, 50 µg/ml), tetracycline (Sigma, 10 µg/ml),or no additional antibiotic. The control cells were washed, re-suspended, and allowed to recover for the same period of time in fresh LB before spotting onto solid media. This experiment was repeated and seven replicate spots from each one-hour time point were analyzed using a 2-way ANOVA test of variance. Results were considered significant if p<0.05, very significant if p<0.01 and extremely significant if p<0.001.

### Cell Morphology Imaging

Cells transformed with pBAD18 plasmids containing either DX, ubiquitin, or the empty vector were grown under induction for 1–9 hours, harvested after 2, 3, 4, 6, 8, and 9 hours of growth, and fixed in 2% gluteraldahyde overnight. These cells were visualized at 40× magnification using a Nikon Eclipse TE300 model microscope using a phase contrast objective. In a second experiment, 100-fold dilutions of overnight growths of cells containing plasmid pBAD18-DX were cultured for 1–8 hours both in the presence and absence of arabinose. These cells were harvested each hour and fixed in 2% gluteraldahyde overnight before imaging between 10 and 20 fields of view for each sample at 40× magnification using a Fisher Micromaster phase contrast microscope. These images were processed using MATLAB (R2008a, The MathWorks) to determine median cell lengths and areas.

### Electron Microscopy

Overnight cultures containing pBAD18 plasmid with the DX insert were diluted 100-fold into liquid media either containing or devoid of arabinose inducer. Liquid cultures were allowed to grow for 3, 4, or 5 hours before being fixed in 2% gluteralehyde in phosphate buffered saline (PBS) (100 mM NaPO_4_ (pH 7.0) and 100 mM NaCl). Cells were washed twice, re-suspended in PBS, and embedded in 1% agarose. Agarose pellets were washed twice in PBS and post-fixed in 1% osmium tetroxide in PBS for 2 hours. Pellets were then washed twice in distilled water (diH_2_O), left in diH_2_O at 4°C overnight, washed twice again in diH_2_O, incubated in 1% uranyl acetate for 2 h, and washed 4x in diH_2_O. Cells were dehydrated by incubating in an ascending series (20% increments) of 10 min acetone washes with three final wash steps in 100% acetone. Dehydrated cells were then infiltrated with Spurr's epoxy resin on a rotating wheel using an ascending series similar to acetone (25% increments) with three final washes in 100% resin. Cells were embedded in fresh resin and polymerized for 24 h at 60°C. 60 nm section were cut using a Leica Ultracut-R microtome and thin sections were post-stained for 5 min with 1% uranyl acetate and 3 min with Sato's lead citrate. Sections were imaged with a Philips CM12 operated at 80 kV and images were generated with a Gatan 791 CCD camera.

### Microarray Analysis

Overnight cultures of cells containing the pBAD18-DX plasmid were diluted 25-fold into media either with or without arabinose inducer. Aliquots were taken every 30 min from time 0 until 4 hours post-induction. Total RNA was extracted using an Invitrogen PureLink Micro-to-Midi RNA purification kit. RNA was analyzed using an Agilent 2100 Bioanalyzer and RNA samples with a RNA integrity number (RIN) of less than 7 were excluded and repeated. cDNA was generated with a FairPlay III microarray labeling kit using random primers. cDNA was purified using an Invitrogen PureLink PCR purification kit and labeled with Molecular Probes Alexa-647 (for RNA from induced cells) or Alexa-555 (for RNA from uninduced cells) following the manufacturer's protocol. Labeled cDNA from each of the eight time points was hybridized to a microarray slide containing 8 separate copies of the *E. coli* genome (Agilent Microarray Design ID 020097) according to instructions provided by the manufacturer. Slides were imaged using an Agilent G2565 BA scanner. Expression data was extracted using GenePix Pro v6.0 software and the provided GAL file. Expression data was analyzed using GeneSpring (Agilent) and MATLAB's bioinfomatics software suit (see supplementary information). Data from the 2.5 hours post-induction sample were excluded from this analysis because the uninduced RNA failed to label properly. Microarray data was deposited in the NCBI microarray database Gene Expression Omnibus with the accession code GSE17568.

## Supporting Information

Figure S1 Colonies grown from cells expressing DX show a distinct morphological phenotype which increases in severity as the as the length of induction time increases. Maintenance of DX expression on solid media also increases the severity of this phenotype.
(1.98 MB TIF)

Figure S2 CFU counts of DX-expressing and control cells during eight hours of induction and recovery. Each time point represents the average number of CFUs obtained from 7–10 µL spots and the error bars represent the standard deviation.
(3.93 MB TIF)

Table S1 CFU counts of cultures expressing DX
(0.06 MB PDF)

Table S2 Genes identified by linear regression as having increased expression over time.
(0.15 MB PDF)

Table S3 Genes identified by linear regression as having decreased expression over time.
(0.13 MB PDF)

Table S4 Time dependent expression of chemotaxis genes.
(0.09 MB PDF)

Table S5 Time dependent expression of sulfur metabolism genes.
(0.07 MB PDF)

Table S6 Time dependent expression of the rpoS regulon.
(0.08 MB PDF)

Table S7 Time dependent expression of SOS pathway genes.
(0.05 MB PDF)

Table S8 Time dependent expression of cell division genes.
(0.10 MB PDF)

Table S9 Expression over time of genes involved in ATP biosynthesis.
(0.10 MB PDF)
