# Supplementary figures and images for: A Man-Made ATP-Binding Protein Evolved Independent of Nature Causes Abnormal Growth in Bacterial Cells
Source: PLoS One. 2009 Oct 8;4(10):e7385. doi: 10.1371/journal.pone.0007385 (PMC2754611; doi:10.1371/journal.pone.0007385)

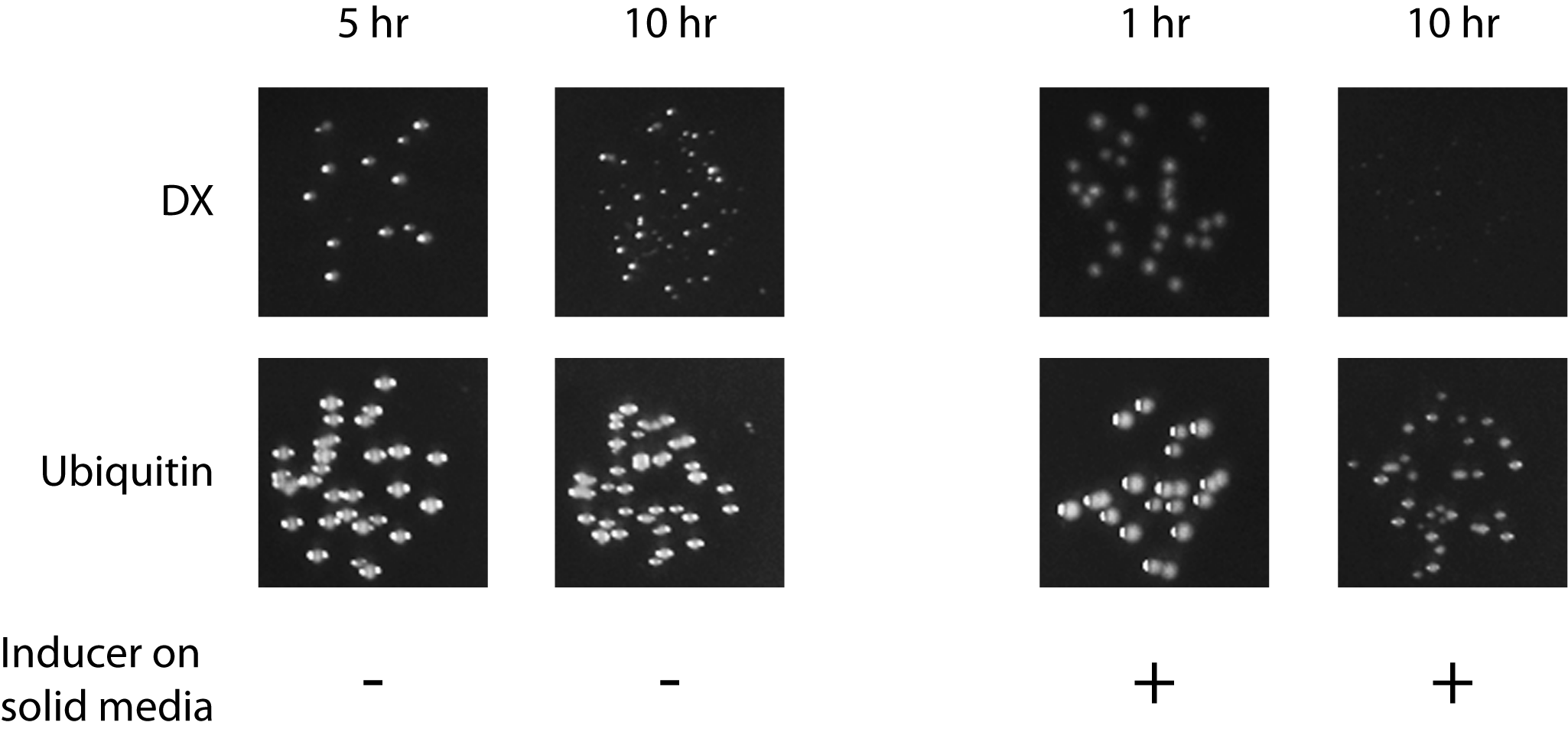

Supplement: Figure S1 — Colonies grown from cells expressing DX show a distinct morphological phenotype which increases in severity as the as the length of induction time increases. Maintenance of DX expression on solid media also increases the severity of this phenotype. (1.98 MB TIF) [file pone.0007385.s001.tif]

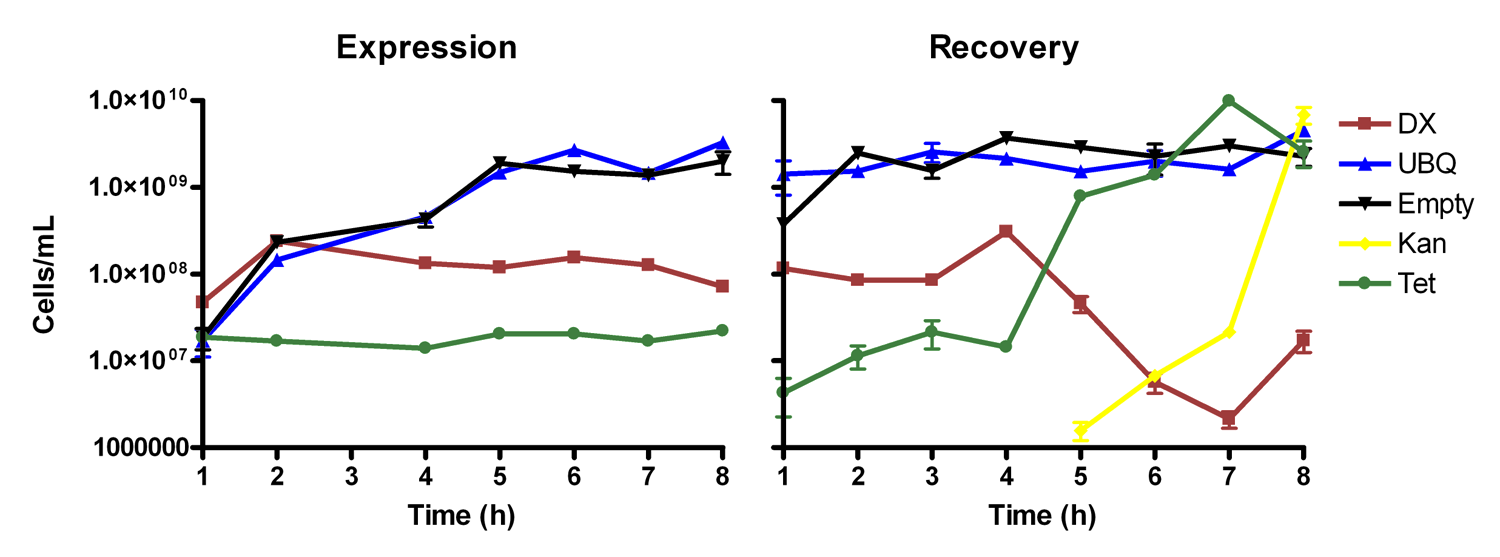

Supplement: Figure S2 — CFU counts of DX-expressing and control cells during eight hours of induction and recovery. Each time point represents the average number of CFUs obtained from 7–10 µL spots and the error bars represent the standard deviation. (3.93 MB TIF) [file pone.0007385.s002.tif]
