# Supplementary material for: A Man-Made ATP-Binding Protein Evolved Independent of Nature Causes Abnormal Growth in Bacterial Cells
Source: PLoS One. 2009 Oct 8;4(10):e7385. doi: 10.1371/journal.pone.0007385 (PMC2754611; doi:10.1371/journal.pone.0007385)
Supplement: Table S1 — CFU counts of cultures expressing DX (0.06 MB PDF) [file pone.0007385.s003.pdf]

	DX		Ubiquitin		Empty		Kan		Tet	
	Mean cells/mL	Standard Deviation	Mean cells/mL	Standard Deviation	Mean cells/mL	Standard Deviation	Mean cells/mL	Standard Deviation	Mean cells/mL	Standard Deviation
	**Expression**									
**1**	4.71E+07	1.70E+07	1.71E+07	1.60E+07	1.86E+07	1.35E+07	0.00E+00	0.00E+00	0.00E+00	4.27E+06
**2**	2.40E+08	5.32E+07	1.46E+08	2.51E+07	2.33E+08	5.65E+07	0.00E+00	0.00E+00	1.69E+07	2.54E+06
**3**	1.31E+08	3.80E+07	4.57E+08	3.31E+08	1.86E+08	6.90E+07	0.00E+00	0.00E+00		
**4**	1.33E+08	4.23E+07	4.57E+08	1.90E+08	4.29E+08	2.06E+08	0.00E+00	0.00E+00	1.40E+07	4.16E+06
**5**	1.20E+08	1.73E+07	1.49E+09	4.10E+08	1.90E+09	4.65E+08	0.00E+00	0.00E+00	2.06E+07	7.09E+06
**6**	1.56E+08	4.79E+07	2.70E+09	3.37E+08	1.53E+09	3.82E+08	0.00E+00	0.00E+00	2.06E+07	2.64E+06
**7**	1.27E+08	3.20E+07	1.47E+09	3.73E+08	1.39E+09	4.56E+08	0.00E+00	0.00E+00	1.69E+07	4.60E+06
**8**	7.14E+07	2.04E+07	3.30E+09	6.51E+08	2.00E+09	2.00E+09	0.00E+00	0.00E+00	2.21E+07	4.71E+06
	**Recovery**									
**1**	1.20E+08	3.56E+07	1.43E+09	1.62E+09	1.62E+09	6.90E+07	0.00E+00	0.00E+00	4.29E+06	5.35E+06
**2**	8.57E+07	3.69E+07	1.54E+09	3.78E+08	2.49E+09	6.69E+08	0.00E+00	0.00E+00	1.14E+06	9.00E+05
**3**	8.67E+07	1.51E+07	2.57E+09	1.72E+09	1.57E+09	7.87E+08	2.86E+05	4.88E+05	2.14E+07	2.04E+07
**4**	3.09E+08	5.96E+07	2.16E+09	3.78E+08	3.80E+09	1.92E+09	1.43E+05	3.78E+05	1.44E+07	1.40E+06
**5**	4.57E+07	2.51E+07	1.53E+09	2.36E+08	2.90E+09	3.42E+08	1.57E+06	9.76E+05	7.90E+08	8.52E+07
**6**	5.71E+06	3.99E+06	2.00E+09	1.73E+09	2.29E+09	2.36E+09	6.71E+06	2.50E+06	1.40E+09	2.31E+08
**7**	2.14E+06	1.21E+06	1.61E+09	4.53E+08	3.00E+09	1.00E+09	2.14E+07	3.31E+06	9.89E+09	1.31E+09
**8**	1.71E+07	1.25E+07	4.59E+09	7.06E+08	2.29E+09	1.38E+09	7.71E+07	2.69E+07	2.57E+09	2.30E+09
