# Supplementary material for: A Man-Made ATP-Binding Protein Evolved Independent of Nature Causes Abnormal Growth in Bacterial Cells
Source: PLoS One. 2009 Oct 8;4(10):e7385. doi: 10.1371/journal.pone.0007385 (PMC2754611; doi:10.1371/journal.pone.0007385)
Supplement: Table S2 — Genes identified by linear regression as having increased expression over time. (0.15 MB PDF) [file pone.0007385.s004.pdf]

Gene	Hours post induction (log_2(induced / un-induced))							Gene Function
	0.5	1	1.5	2	3	3.5	4	
ACA-0244248	-0.09	-0.33	-0.27	-0.15	0.03	0.40	0.34	transposase Tra5 []
allB	-0.35	-0.45	0.12	0.24	0.81	1.68	1.51	putative hydrolase [b0512]
alpA	-0.09	-0.04	0.37	0.10	0.45	1.10	1.11	Prophage CP4-57 Regulatory protein alpA [c_1169]
amiC	-0.23	-0.09	0.68	0.68	1.28	1.62	1.52	putative amidase [b2817]
b2649	0.29	0.29	0.60	0.75	0.27	1.34	1.69	"orf, hypothetical protein [b2649]"
b2656	-0.10	0.02	0.20	-0.14	0.43	0.84	0.82	"orf, hypothetical protein [b2656]"
bioC	-0.06	-0.17	0.06	-0.11	0.01	0.53	0.40	biotin biosynthesis; reaction prior to pimeloyl CoA [b0777]
c_0273	-0.15	-0.05	-0.20	-0.22	0.00	0.81	0.39	Putative radC-like protein yeeS [c_0273]
c_0429	-0.17	-0.24	-0.01	0.42	-0.21	0.35	-0.01	Conserved hypothetical protein [c_0429]
c_0438	-0.05	-0.01	-0.81	-0.42	-0.08	-0.25	-0.18	Hypothetical protein [c_0438]
c_0471	0.00	0.00	-0.02	-0.21	0.05	0.92	0.73	Hypothetical protein [c_0471]
c_1145	0.01	-0.19	0.03	-0.13	0.31	0.23	0.56	Putative NADH dehydrogenase/NAD [c_1145]
c_1883	0.05	-0.46	-0.26	0.22	-0.03	0.17	-0.03	Conserved hypothetical protein [c_1883]
c_1891	0.02	-0.10	0.10	0.17	-0.07	0.34	0.05	Hypothetical protein [c_1891]
c_2118	0.00	-0.19	0.05	0.01	0.07	0.90	1.06	Putative conserved protein [c_2118]
c_2180	-0.10	0.03	-0.17	-0.14	-0.02	0.38	0.29	Hypothetical protein [c_2180]
c_2442	-0.02	-0.38	-0.16	-0.09	0.04	0.27	0.37	Hypothetical protein [c_2442]
c_2456	0.18	-0.31	-0.03	0.06	-0.08	0.22	0.00	Hypothetical protein [c_2456]
c_2489	0.01	-0.01	-0.03	-0.05	0.13	0.45	0.37	Putative transferase [c_2489]
c_2525	-0.07	-0.31	-0.22	-0.07	-0.10	0.22	-0.14	Hypothetical protein [c_2525]
c_2924	-0.10	-0.05	-0.03	-0.26	0.03	0.78	0.48	Putative peptidase ypdF [c_2924]
c_3166	-0.02	-0.28	-0.12	0.06	-0.12	0.26	0.03	Putative head-tail joining protein of prophage [c_3166]
c_3559	-0.09	0.09	0.28	0.17	0.01	0.38	0.23	Hypothetical protein [c_3559]
c_3631	0.05	-0.59	0.48	0.72	0.08	0.11	-0.03	Hypothetical protein [c_3631]
c_4435	-0.54	-0.47	-0.26	-0.25	0.00	0.41	0.57	Hypothetical protein [c_4435]
c_4576	-0.04	-0.14	0.05	-0.04	0.11	0.61	0.44	Hypothetical protein yeeV [c_4576]
c_4808	-0.17	-0.77	0.45	0.29	1.15	2.06	2.21	Hypothetical protein [c_4808]
c_5447	0.08	-0.17	1.77	1.60	2.21	2.86	3.60	Hypothetical protein [c_5447]
c_5448	0.16	-0.01	1.53	1.73	2.71	3.45	3.60	Hypothetical protein [c_5448]
caiD	-0.01	-0.38	-0.13	-0.16	0.66	1.27	1.49	carnitine racemase [b0036]
cheB	-0.06	-0.24	-0.01	-0.14	0.22	0.25	0.36	response regulator for chemotaxis [b1883]
cheZ	-0.30	-0.02	-0.08	-0.23	0.72	0.66	0.55	Chemotaxis protein cheZ [c_2296]
chpR	-0.04	0.15	0.95	0.68	0.86	1.61	1.77	"suppressor of inhibitory function of ChpA, PemI-like, autoregulated [b2783]"
citG	0.01	0.03	0.03	0.12	-0.08	0.68	0.93	"orf, hypothetical protein [b0613]"
cpsG	-0.06	0.02	0.14	-0.16	0.21	0.54	0.35	phosphomannomutase [b2048]

csgC	-0.08	-0.25	-0.03	0.05	0.23	0.43	0.76	putative curli production protein [b1043]
cspF	-0.37	-0.64	1.97	1.79	2.74	3.92	4.04	cold shock protein [b1558]
cspH	0.12	-0.33	2.00	1.93	2.51	3.54	3.58	cold shock-like protein [b0989]
cysH	0.01	-0.21	-0.16	-0.04	0.04	0.19	0.15	3-phosphoadenosine 5-phosphosulfate reductase [b2762]
cysJ	0.16	-0.22	-0.17	-0.05	0.39	-0.33	0.14	Sulfite reductase [c_3323]
cysN	-0.04	-0.24	-0.18	-0.08	-0.06	0.14	0.15	ATP-sulfurylase [b2751]
DCP_22_7	-0.09	-0.47	-0.14	0.12	-0.06	0.15	-0.04	
dpiB	0.11	-0.27	0.03	-0.10	-0.02	0.49	0.71	putative sensor-type protein [b0619]
ECs0325	0.18	-0.18	0.73	0.57	1.23	1.95	1.86	hypothetical protein [ECs0325]
ECs0331	0.11	0.01	0.25	0.01	-0.13	1.25	1.92	putative NADH-dependent flavin oxidoreductase [ECs0331]
ECs0337	-0.10	-0.23	0.04	-0.29	-0.18	0.70	0.44	putative transcription regulator [ECs0337]
ECs0573	0.03	-0.15	0.15	0.22	1.21	1.36	1.70	allantoinase [ECs0573]
ECs0854	0.06	-0.09	0.09	0.11	0.37	0.42	0.42	8-amino-7-oxononanoate synthase [ECs0854]
ECs1613	0.04	0.19	1.11	1.46	1.60	2.92	2.73	Ren protein [ECs1613]
ECs1628	-0.68	-0.20	0.32	0.13	0.81	0.90	1.34	hypothetical protein [ECs1628]
ECs1721	0.20	0.39	1.13	2.22	2.54	2.42	2.76	sodium-calcium/proton antiporter [ECs1721]
ECs1946	-0.16	0.06	0.17	0.13	0.23	0.84	0.51	hypothetical protein [ECs1946]
ECs2038	0.01	-0.75	-0.68	-0.52	0.24	0.16	1.20	hypothetical protein [ECs2038]
ECs2427	-0.12	0.01	-0.12	0.03	-0.08	0.52	0.50	hypothetical protein [ECs2427]
ECs2928	-0.16	-0.39	-0.10	-0.24	0.21	0.54	0.61	hypothetical protein [ECs2928]
ECs2985	0.41	0.30	1.25	1.57	2.37	2.80	2.99	Ren protein [ECs2985]
ECs3484	0.01	-0.11	0.69	0.43	1.83	2.29	2.71	hypothetical protein [ECs3484]
ECs3559	-0.35	-0.70	0.91	2.63	4.01	4.80	5.31	interrupted glucitol/sorbitol-specific PTS system enzyme IIC component [ECs3559]
ECs3592	0.16	-0.37	-0.11	0.20	0.16	0.06	0.06	putative 4-hydroxybenzoate decarboxylase [ECs3592]
ECs3631	-0.12	-0.19	-0.53	-0.23	0.18	0.61	0.48	putative transport protein [ECs3631]
ECs4471	0.23	0.04	0.84	0.65	1.79	2.60	3.24	hypothetical protein [ECs4471]
ECs4472	-0.04	0.40	0.47	0.35	0.89	1.71	1.92	hypothetical protein [ECs4472]
ECs4500	0.01	-0.35	-0.19	0.46	-0.10	0.16	0.03	lipid A-core:surface polymer ligase [ECs4500]
ECs4630	0.01	-0.09	0.07	-0.10	0.07	0.61	0.61	hypothetical protein [ECs4630]
ECs5021	-0.18	-0.57	-0.19	-0.14	0.18	0.68	0.74	hypothetical protein [ECs5021]
ECs5058	0.06	-0.28	0.16	-0.01	0.16	0.48	0.65	part of formate-dependent nitrite reductase complex NrfG [ECs5058]
ECs5233	-0.23	-0.20	-0.12	-0.14	-0.23	0.56	0.12	hypothetical protein [ECs5233]
eutE	-0.07	-0.11	0.08	-0.23	0.23	0.44	0.55	ethanolamine utilization; similar to acetaldehyde dehydrogenase [b2455]
flgD	-0.07	-0.01	0.03	-0.55	0.10	0.48	0.23	"flagellar biosynthesis, initiation of hook assembly [b1075]"
flgE	-0.11	-0.16	-0.14	-0.33	0.13	0.23	0.28	"flagellar biosynthesis, hook protein [b1076]"
flgJ	0.00	0.06	0.03	-0.27	-0.05	0.49	0.25	flagellar biosynthesis [b1081]
flhE	-0.03	-0.05	0.25	0.02	0.37	0.69	0.77	flagellar protein [b1878]

fliP	-0.01	-0.13	0.03	-0.19	0.28	0.56	0.51	Flagellar biosynthetic protein fliP precursor [c_2365]
frwB	-0.27	-0.88	-0.24	-0.07	0.31	0.63	0.67	PTS system fructose-like IIB component 1 [b3950]
ggt	0.02	-0.25	-0.42	-0.40	-0.26	0.00	-0.14	gamma-glutamyltranspeptidase [Z4813]
gnd	-0.99	-0.69	-0.80	-0.68	-0.02	0.78	0.98	"gluconate-6-phosphate dehydrogenase, decarboxylating [b2029]"
hha	-0.10	-0.45	1.02	1.15	2.61	3.28	3.53	haemolysin expression modulating protein [b0460]
hofP	-0.03	-0.15	-0.09	-0.08	0.10	0.43	0.65	"orf, hypothetical protein [b3392]"
hyfA	0.00	0.06	-0.09	-0.26	0.01	0.46	0.48	hydrogenase 4 Fe-S subunit [b2481]
iscS	0.23	0.33	0.47	0.97	1.93	2.45	2.63	putative aminotransferase [b2530]
iscU	-0.31	0.29	0.65	0.92	1.07	2.26	2.21	"orf, hypothetical protein [b2529]"
ispU	-0.20	-0.35	0.31	0.79	0.63	1.47	1.45	"orf, hypothetical protein [b0174]"
kdpC	-0.03	-0.13	0.10	-0.02	0.38	0.85	1.01	high-affinity potassium transport system [b0696]
kil	0.02	-0.05	0.23	0.11	0.19	0.91	1.45	hypothetical protein [ECs1936]
kilR	-0.02	-0.22	0.22	0.17	0.50	1.07	1.23	"orf, hypothetical protein [b1352]"
L7026	0.06	-0.32	0.16	0.37	0.00	0.12	0.00	polysaccharide deacetylase [L7026]
lar	0.10	0.20	1.21	0.73	2.58	3.30	3.70	restriction alleviation and modification enhancement protein [ECs1932]
livM	-0.05	-0.45	-0.03	-0.05	0.04	0.80	0.91	high-affinity branched-chain amino acid transport [b3456]
lsrC	0.01	-0.30	-0.16	-0.18	-0.01	0.35	0.74	putative transport system permease protein [b1514]
maa	0.37	0.19	1.84	1.97	3.26	3.79	3.99	putative transferase [b0459]
maoC	0.06	-0.28	0.02	0.01	0.26	0.68	0.66	putative aldehyde dehydrogenase [b1387]
mdtI	0.39	0.07	0.76	1.36	1.94	2.87	2.89	possible chaperone [b1599]
mhpT	-0.10	-0.06	0.08	-0.16	0.04	0.84	0.70	putative transport protein [b0353]
mmuP	-0.23	-0.48	-0.09	-0.13	0.23	1.04	1.00	putative amino acid/amine transport protein [b0260]
nrfG	0.06	-0.36	0.30	0.03	0.25	0.56	0.58	part of formate-dependent nitrite reductase complex [b4076]
nsrR	-0.09	-0.11	0.36	0.67	1.19	1.75	1.79	"orf, hypothetical protein [b4178]"
ompF	-0.73	-1.02	-0.04	0.35	0.87	1.01	1.79	outer membrane protein 1a [b0929]
ompG	0.04	-0.09	0.33	0.15	0.53	0.93	1.00	outer membrane protein [Z2462]
pgaC	0.06	-0.06	0.87	0.60	1.48	1.56	1.98	"orf, hypothetical protein [b1022]"
pphA	-0.33	-0.33	-0.13	-0.12	0.74	1.18	1.75	"protein phosphatase 1 modulates phosphoproteins, signals protein misfolding [b1838]"
puuC	0.05	-0.11	0.09	-0.30	0.30	0.42	0.75	"aldehyde dehydrogenase, prefers NADP over NAD [b1300]"
puuE	-0.03	-0.02	0.13	-0.28	0.13	0.59	0.73	4-aminobutyrate aminotransferase [b1302]
rarD	-0.19	-0.20	0.33	0.12	-0.12	0.76	0.78	"orf, hypothetical protein [b3819]"
rdoA	-0.23	-0.36	-0.11	0.15	0.44	1.51	1.92	"orf, hypothetical protein [b3859]"
recE	0.06	0.03	0.22	0.28	0.64	0.83	0.94	Exodeoxyribonuclease VIII [c_1402]
recT	-0.07	-0.07	1.34	0.79	2.07	2.93	3.25	"recombinase, DNA renaturation [b1349]"
rpmE2	-0.12	0.72	1.62	1.55	2.08	2.76	3.30	ribosomal protein L31-like protein [ECs1330]
rsxA	0.01	0.17	0.44	0.81	0.99	2.29	2.13	"orf, hypothetical protein [b1627]"
sgbH	-0.03	-0.25	-0.09	-0.29	0.05	0.52	0.35	probable 3-hexulose 6-phosphate synthase [b3581]

sgbU	-0.01	-0.18	-0.04	-0.31	0.35	0.52	0.54	Putative hexulose-6-phosphate isomerase [c_4405]
srlA_1	-0.76	-0.58	0.72	3.55	3.85	4.81	5.42	"PTS system, glucitol/sorbitol-specific IIC component, one of [Z4005]"
tauD	-0.03	-0.20	0.08	0.01	0.04	0.42	0.38	"taurine dioxygenase, 2-oxoglutarate-dependent [Z0467]"
tdcR	-0.15	-0.35	-0.01	-0.18	0.91	0.80	1.56	threonine dehydratase operon activator protein [Z4471]
tfaR	0.06	-0.13	0.27	0.29	0.86	1.04	1.37	"orf, hypothetical protein [b1373]"
tfaX	-0.30	0.06	1.31	0.91	2.45	2.83	3.74	"orf, hypothetical protein [b0563]"
thiG	0.00	-0.30	-0.25	-0.36	-0.20	0.26	0.17	"thiamin biosynthesis, thiazole moiety [b3991]"
torA	-0.01	-0.32	-0.02	-0.29	-0.03	0.34	0.03	Trimethylamine-N-oxide reductase 1 precursor [c_1133]
torI	-0.85	-0.93	-0.93	-0.54	0.56	1.29	1.10	response regulator inhibitor for tor operon [b4501]
ubiB	-0.78	-0.62	-0.32	-0.08	-0.28	0.21	0.34	"orf, hypothetical protein [b3835]"
ugd	0.27	0.21	0.49	0.57	0.98	1.17	1.07	UDP-glucose 6-dehydrogenase [b2028]
ulaA	-0.02	-0.05	-0.04	-0.09	0.00	0.26	0.23	orf Unknown function [Z1087]
wcaA	-0.07	-0.16	0.00	-0.24	0.25	0.36	0.37	putative regulator [b2059]
wcaB	0.01	-0.32	-0.05	-0.23	0.08	0.55	0.45	putative transferase [Z3222]
wcaD	0.03	-0.16	0.48	0.49	0.27	1.53	1.63	putative colanic acid polymerase [b2056]
wcaM	0.02	0.17	0.85	0.66	1.08	1.46	1.37	"orf, hypothetical protein [b2043]"
xapR	0.21	-0.14	0.32	0.44	0.33	0.47	0.37	Xanthosine operon Regulatory protein [c_2937]
yagK	-0.06	-0.45	0.86	0.74	2.40	2.60	2.88	"orf, hypothetical protein [b0277]"
ybaJ	-0.27	-0.83	0.63	0.98	2.34	3.26	3.50	"orf, hypothetical protein [b0461]"
ybcV	-0.43	-0.61	0.34	0.11	1.05	1.63	1.91	putative an envelop protein [b0558]
ybcY	-0.29	-0.48	0.64	0.66	0.87	1.80	1.56	"orf, hypothetical protein [b0562]"
ybdB	-0.17	0.15	0.50	0.41	0.64	1.09	1.28	"orf, hypothetical protein [Z0739]"
ybeA	-0.19	-0.41	0.63	0.53	0.83	1.14	1.53	"orf, hypothetical protein [b0636]"
ycaK	-0.70	-0.36	0.35	0.56	0.52	1.29	1.65	"orf, hypothetical protein [b0901]"
ycbX	-0.16	-0.06	0.05	0.26	-0.05	0.72	0.22	"orf, hypothetical protein [b0947]"
ycdO	0.07	0.32	0.25	0.40	0.11	1.07	1.12	Protein ycdO [c_1156]
ycdU	0.28	-0.66	1.54	1.70	2.55	3.51	4.19	"orf, hypothetical protein [b1029]"
ycjO	-0.10	-0.13	-0.28	-0.28	0.34	0.20	0.54	putative binding-protein dependent transport protein [b1311]
ycjP	-0.02	-0.14	-0.17	-0.15	0.42	0.79	0.97	putative transport system permease protein [Z2471]
ycjP	-0.02	-0.14	-0.17	-0.15	0.42	0.79	0.97	putative transport system permease protein [Z2471]
ydaC	-0.02	0.09	1.16	0.77	2.37	3.04	3.42	unknown protein encoded within prophage CP-933R [Z2413]
ydaJ	0.03	-0.19	-0.17	-0.11	0.09	0.40	0.27	putative aminohydrolase [Z2425]
ydbD	0.02	-0.23	0.18	0.26	0.05	1.20	1.23	"orf, hypothetical protein [b1407]"
ydeK	-0.10	-0.55	-0.47	-0.28	-0.28	0.02	0.16	"orf, hypothetical protein [b1510]"
ydeO	0.06	0.25	0.57	0.33	1.03	2.24	2.99	putative ARAC-type regulatory protein [b1499]
ydeP	-0.12	-0.19	0.08	0.21	0.50	1.11	1.25	"putative oxidoreductase, major subunit [b1501]"
ydgK	-0.93	-0.55	0.19	0.36	0.81	2.05	2.71	"orf, hypothetical protein [b1626]"

ydiM	0.07	-0.29	-0.08	-0.06	0.69	0.60	0.95	putative transport system permease protein [b1690]
ydiY	-0.31	-0.23	0.23	0.19	0.12	0.80	0.56	"orf, hypothetical protein [b1722]"
yeaE	0.05	-0.04	-0.82	-0.45	-0.15	-0.08	-0.50	Hypothetical protein yeaE [c_2186]
yebB	0.23	0.02	-0.03	0.33	-0.01	0.04	0.42	Hypothetical protein yebB [c_2276]
yehA	0.05	0.15	0.28	0.16	1.00	1.70	2.01	Hypothetical protein yehA precursor [c_2635]
yeiM	-0.07	-0.44	0.23	0.31	0.32	0.69	0.88	putative transport system permease protein [b2164]
yeiN	-0.05	-0.10	0.06	0.22	0.19	0.35	0.69	"orf, hypothetical protein [b2165]"
yfaV	0.11	-0.41	-0.09	-0.08	0.01	0.51	1.25	putative transport protein [b2246]
yfbL	-0.02	0.19	0.28	0.17	0.09	0.65	0.59	putative aminopeptidase [b2271]
yfcS	0.03	-0.07	0.00	-0.02	0.13	-0.12	0.16	Hypothetical fimbrial chaperone yfcS precursor [c_2882]
yfdM	0.07	-0.14	0.16	0.03	-0.10	0.81	0.64	"orf, hypothetical protein [b2356]"
yfjJ	0.04	-0.13	0.29	0.42	0.66	1.36	2.10	"orf, hypothetical protein [b2626]"
yfjK	-0.50	-0.22	0.25	0.14	0.33	1.30	1.08	"orf, hypothetical protein [b2627]"
ygcG	-0.01	-0.29	0.20	0.05	-0.13	0.28	0.11	"orf, hypothetical protein [b2778]"
ygcY	-0.10	-0.12	-0.63	-0.45	-0.09	0.27	0.25	Glucarate dehydratase related protein [c_3352]
ygdQ	-0.06	-0.16	0.59	0.85	0.82	1.16	1.55	putative transport protein [b2832]
ygjQ	0.01	0.13	0.03	-0.07	0.04	0.42	-0.06	"orf, hypothetical protein [b3086]"
yhjR	0.02	-0.31	0.46	0.90	2.16	2.24	2.27	"orf, hypothetical protein [b3535]"
yiaO	-0.13	-0.04	0.01	-0.38	0.28	0.47	0.71	putative solute-binding transport protein [b3579]
yibG	0.39	-0.14	1.03	0.55	1.40	2.51	3.22	"orf, hypothetical protein [b3596]"
yibJ	-0.39	-0.61	0.25	0.36	0.93	1.56	1.88	"orf, hypothetical protein [b3595]"
yidD	-0.24	0.08	0.16	0.25	0.72	0.83	1.50	predicted protein [b4557]
yieG	0.24	0.06	0.72	1.04	0.78	1.86	1.83	Hypothetical protein yieG [c_4637]
yjcF	-0.02	-0.49	0.16	0.13	0.25	0.94	0.53	"orf, hypothetical protein [b4066]"
yjgZ	-0.12	-0.16	-0.19	-0.20	0.01	0.51	0.37	"orf, hypothetical protein [b4277]"
yjhC	-0.34	-0.80	-0.39	-0.01	2.09	2.84	2.73	putative dehydrogenase [b4280]
yjhE	0.27	0.23	1.21	1.37	1.43	2.05	2.11	"orf, hypothetical protein [b4282]"
ykgM	-0.02	1.20	3.32	2.97	4.21	5.27	5.90	putative ribosomal protein [b0296]
ynbD	-0.10	-0.25	-0.14	-0.02	-0.06	0.40	0.14	putative enzymes [Z2316]
ynfA	0.34	-0.07	0.70	0.99	1.10	0.96	1.40	"orf, hypothetical protein [b1582]"
yoeB	-0.06	0.05	0.35	0.41	0.56	1.26	1.26	toxin of the YoeB-YefM toxin-antitoxin system [b4539]
yohF	0.00	0.09	0.03	0.45	0.39	0.61	0.53	putative oxidoreductase [b2137]
ypdE	-0.12	-0.21	0.00	-0.31	0.23	0.57	0.58	"orf, hypothetical protein [b2384]"
yphC	0.01	-0.36	-0.40	-0.27	-0.35	-0.12	-0.22	Hypothetical zinc-type alcohol dehydrogenase-like protein yphC [c_3067]
yqcE	0.10	-0.23	-0.44	-0.18	0.25	0.47	0.55	putative transport protein [Z4086]
yrbF	-0.22	-0.43	0.26	0.43	0.59	0.97	1.26	putative ATP-binding component of a transport system [b3195]
ytfI	-0.03	-0.66	-0.13	-0.22	1.10	1.81	2.59	"orf, hypothetical protein [b4215]"

Z0390	0.06	-0.25	0.16	0.23	0.04	-0.05	-0.01	orf Unknown function [Z0390]
Z0414	0.11	-0.26	0.04	0.55	1.14	0.26	0.08	orf Unknown function [Z0414]
Z1218	-0.10	-0.20	-0.04	-0.11	0.25	0.38	0.42	"orf, hypothetical protein [Z1218]"
Z1362	0.23	-0.13	-0.01	0.28	-0.04	0.37	0.06	unknown protein encoded by cryptic prophage CP-933M [Z1362]
Z1507	-0.10	-0.08	-0.05	-0.24	0.01	0.63	0.55	putative enzyme [Z1507]
Z2286	-0.08	-0.68	-0.73	-0.50	0.46	0.46	1.12	putative membrane transport protein [Z2286]
Z3306	0.00	-0.26	-0.08	-0.10	-0.14	0.22	0.09	unknown protein encoded within prophage CP-933V [Z3306]
Z4045	-0.09	-0.17	-0.08	0.01	-0.12	0.19	-0.08	orf hypothetical protein [Z4045]
Z4046	-0.03	-0.28	-0.04	0.17	-0.06	0.19	-0.02	orf hypothetical protein [Z4046]
Z4105	-0.12	-0.19	-0.88	-0.87	-0.37	0.33	0.36	putative transport protein [Z4105]
Z5154	0.06	-0.07	0.08	-0.03	0.21	0.47	0.66	orf Unknown function [Z5154]
Z5868	-0.03	-0.25	-0.06	-0.09	0.01	0.27	0.22	orf Unknown function [Z5868]
zwf	-0.18	0.13	0.13	0.19	0.53	0.62	0.77	glucose-6-phosphate dehydrogenase [b1852]
