# Supplementary material for: A Man-Made ATP-Binding Protein Evolved Independent of Nature Causes Abnormal Growth in Bacterial Cells
Source: PLoS One. 2009 Oct 8;4(10):e7385. doi: 10.1371/journal.pone.0007385 (PMC2754611; doi:10.1371/journal.pone.0007385)
Supplement: Table S3 — Genes identified by linear regression as having decreased expression over time. (0.13 MB PDF) [file pone.0007385.s005.pdf]

Gene	Hours post induction ($\log_2$(induced / un-induced))							Gene Function
	0.5	1	1.5	2	3	3.5	4	
acs	0.07	-0.23	0.19	0.27	0.09	-1.35	-1.99	acetyl-CoA synthetase [Z5668]
actP	0.01	-0.18	0.10	-0.01	0.17	-0.94	-1.46	putative transport protein [b4067]
ada	0.14	-0.47	-0.52	-0.35	-0.56	-1.21	-1.42	ADA Regulatory protein [c_2754]
aidB	0.21	-0.11	-0.80	-1.19	-1.00	-1.82	-2.00	putative acyl coenzyme A dehydrogenase [b4187]
allR	0.03	-0.47	-0.63	-0.69	-1.27	-1.71	-2.13	putative regulator [b0506]
ansB	-0.19	-0.36	-1.73	-1.38	0.00	-2.85	-3.65	periplasmic L-asparaginase II [b2957]
appA	-0.01	-0.21	-0.94	-1.13	-1.28	-1.46	-2.35	Periplasmic appA protein precursor [c_1121]
artJ	-0.20	-0.18	-0.16	-0.55	-0.52	-0.47	-0.72	Arginine-binding periplasmic protein 2 precursor [c_0993]
artM	0.01	-0.48	-1.03	-0.81	-1.15	-1.87	-1.53	arginine 3rd transport system permease protein [ECs0944]
artQ	-0.18	-0.21	-1.01	-0.93	-1.67	-1.91	-1.80	arginine 3rd transport system permease protein [b0862]
atoC	-0.19	-0.39	-0.84	-0.60	-0.85	-0.99	-1.35	"response regulator of ato, ornithine decarboxylase antizyme [b2220]"
b0057	0.23	0.12	-0.12	-0.36	-0.14	-0.24	-0.52	"orf, hypothetical protein [b0057]"
bcsC	0.04	-0.47	-0.90	-1.04	-0.83	-1.86	-1.77	putative oxidoreductase subunit [b3530]
c_0226	0.30	0.01	0.05	-0.04	-0.67	-0.50	-1.10	Putative cell cycle protein mesJ [c_0226]
c_0334	0.10	-0.14	-0.14	-0.05	-0.10	-0.04	-0.40	Putative integral membrane protein [c_0334]
c_0887	-0.02	0.35	-0.13	-0.37	-0.68	-1.54	-2.14	Hypothetical protein [c_0887]
c_0888	-0.07	-0.01	-0.15	-0.25	-0.65	-1.31	-2.81	Hypothetical protein ybiI [c_0888]
c_0955	-0.09	0.73	-0.04	0.00	-0.26	0.13	-0.13	Probable phage tail protein [c_0955]
c_0957	0.97	0.58	-0.03	0.06	-0.43	-0.69	-0.69	Fels-2 prophage: probable prophage lysozyme [c_0957]
c_1498	1.69	1.00	0.39	0.43	0.08	-0.63	-0.78	Hypothetical protein [c_1498]
c_1540	0.02	0.94	0.07	-0.14	-0.21	0.12	-0.03	Lambda Regulatory protein CIII [c_1540]
c_1752	-0.12	-0.28	-0.81	-1.23	-1.23	-3.40	-3.17	Hypothetical protein [c_1752]
c_1810	0.24	0.48	0.19	0.19	-0.04	0.02	0.04	Hypothetical protein [c_1810]
c_1880	0.03	0.10	-0.25	-0.33	-0.86	-1.61	-1.89	Putative conserved protein [c_1880]
c_2145	0.22	1.52	0.16	0.12	-0.06	-0.36	-0.94	Succinylarginine dihydrolase [c_2145]
c_2466	-0.03	0.48	0.24	0.19	-0.14	0.02	-0.03	Hypothetical protein [c_2466]
c_2520	0.01	0.84	0.05	0.33	0.33	0.26	-0.64	Conserved hypothetical protein [c_2520]
c_2806	-0.58	-0.73	-0.67	-0.90	-1.02	-1.47	-1.66	Hypothetical protein [c_2806]
c_4763	0.06	0.07	-0.06	0.07	-0.08	0.08	0.12	Hypothetical protein [c_4763]
c_4977	0.01	1.20	-0.06	0.30	0.02	0.10	0.10	Hypothetical protein [c_4977]
c_5212	-0.07	0.10	0.08	0.13	-0.24	0.13	0.03	Hypothetical protein [c_5212]
c_5381	-0.05	0.20	0.12	-0.14	-0.38	0.08	-0.82	Hypothetical protein [c_5381]
cbpA	-0.14	-0.30	-2.07	-2.45	-2.65	-2.91	-3.25	curved DNA-binding protein; functions closely related to DnaJ [b1000]
cca	0.14	-0.59	-0.55	-0.34	-0.66	-0.72	-1.06	tRNA nucleotidyltransferase [c_3806]
chaC	-0.09	-0.10	-0.18	-0.48	-0.58	-0.54	-0.87	cation transport regulator [b1218]

cspD	-1.42	-1.47	-2.02	-2.19	-2.57	-2.90	-3.54	cold shock protein [b0880]
cueR	-0.02	0.14	-0.52	-1.58	-2.19	-2.77	-2.95	putative transcriptional regulator [b0487]
cysK	-0.42	-0.30	-1.14	-1.48	-1.92	-2.07	-2.02	"cysteine synthase A, O-acetylserine sulfhydrolase A [b2414]"
cysQ	-0.05	-0.37	-0.65	-0.82	-0.72	-0.75	-1.23	affects pool of 3-phosphoadenosine-5-phosphosulfate in pathway of sulfite synthesis [b4214]
dapB	-0.10	-0.13	-0.60	-0.90	-0.72	-1.04	-0.99	dihydrodipicolinate reductase [b0031]
deoB	-0.03	0.17	-0.63	-0.79	-1.14	-1.43	-1.33	phosphopentomutase [b4383]
dkgA	-0.28	-0.17	-1.34	-1.71	-1.67	-1.85	-2.53	"orf, hypothetical protein [b3012]"
ECs0076	0.57	0.40	-0.61	-0.52	-0.72	-1.19	-1.24	3-isopropylmalate isomerase [ECs0076]
ECs0329	0.67	0.89	-0.01	0.34	-0.64	-0.29	-0.90	hypothetical protein [ECs0329]
ECs1335	0.01	0.27	0.02	0.13	-0.16	0.21	-0.56	hypothetical protein [ECs1335]
ECs1478	0.02	0.08	-0.85	-1.07	-0.71	-1.96	-2.05	hypothetical protein [ECs1478]
ECs1634	-0.05	0.92	-0.03	0.07	-0.10	0.06	-0.14	major capsid protein [ECs1634]
ECs1975	2.08	1.65	0.91	0.76	0.21	0.07	-0.12	hypothetical protein [ECs1975]
ECs2304	0.26	-0.41	-0.31	-0.58	-1.17	-0.74	-1.07	hypothetical protein [ECs2304]
ECs2325	-0.07	0.51	-0.91	-0.93	-1.25	-2.33	-2.13	interrupted beta-D-glucuronidase [ECs2325]
ECs2373	-0.10	0.47	-0.36	-0.63	-1.04	-1.66	-1.97	possible enzyme [ECs2373]
ECs2800	0.09	0.82	0.16	0.09	0.12	-0.71	0.12	hypothetical protein [ECs2800]
ECs2888	-0.01	-0.66	-1.80	-2.67	-3.31	-4.42	-4.00	hypothetical protein [ECs2888]
ECs3029	0.90	0.61	0.31	0.29	-0.66	-1.38	-1.33	putative isomerase [ECs3029]
ECs3121	0.01	-0.05	0.04	0.01	-0.18	0.09	-0.07	putative antibiotic resistance protein [ECs3121]
ECs3214	0.05	-0.02	-0.08	-0.20	-0.37	-0.02	0.03	hypothetical protein [ECs3214]
ECs3498	0.30	0.45	0.24	0.14	-0.27	0.04	-0.17	hypothetical protein [ECs3498]
ECs3610	0.15	0.42	0.01	0.05	-0.19	0.16	-0.20	hypothetical protein [ECs3610]
ECs4542	0.07	0.74	-0.09	0.24	-0.21	0.25	-0.14	hypothetical protein [ECs4542]
ECs4548	0.03	1.07	0.62	0.35	0.13	-0.09	-0.01	hypothetical protein [ECs4548]
ECs4668	0.05	0.07	0.04	0.05	-0.10	0.13	0.06	hypothetical protein [ECs4668]
ECs4847	-0.49	-0.27	-1.38	-1.79	-1.75	-2.32	-2.24	hypothetical protein [ECs4847]
ECs4848	-0.20	-0.37	-1.38	-2.17	-1.91	-2.30	-2.30	putative regulator [ECs4848]
ECs4961	-0.02	0.68	0.00	0.15	0.04	0.17	-0.03	putative transcription regulator [ECs4961]
ECs4968	-0.05	0.56	0.20	0.01	-0.16	0.24	-0.13	hypothetical protein [ECs4968]
ECs4980	1.52	1.13	0.00	0.62	-0.46	-0.29	-0.57	hypothetical protein [ECs4980]
ECs4988	0.01	-0.03	-0.10	-0.05	-0.18	-0.46	-0.55	hypothetical protein [ECs4988]
ECs5051	0.01	-0.04	0.11	-0.01	-0.39	-1.10	-2.31	acetyl-CoA synthetase [ECs5051]
erfK	0.03	-0.62	-1.27	-0.88	-1.08	-1.64	-2.03	Protein erfK/srfK precursor [c_2476]
flgN	-0.33	-0.23	-0.87	-0.80	-0.89	-0.89	-1.35	protein of flagellar biosynthesis [b1070]
focF	0.00	0.30	-0.13	0.08	0.21	-0.57	-0.03	F1C minor fimbrial subunit F precursor [c_1243]

frr	-0.12	0.00	-0.16	-0.35	-0.54	-0.72	-1.04	ribosome releasing factor [ECs0174]
galM	-0.04	0.00	-0.85	-0.63	-1.15	-1.03	-1.50	galactose-1-epimerase [b0756]
gcvP	0.00	-0.16	-1.05	-1.17	-1.04	-1.13	-1.47	"glycine decarboxylase, P protein of glycine cleavage [Z4240]"
gst	-0.12	0.36	-0.23	-0.90	-1.62	-1.61	-1.92	glutathionine S-transferase [b1635]
hdeD	-0.07	-0.68	-1.83	-2.10	-1.97	-3.58	-3.59	"orf, hypothetical protein [b3511]"
hisI	-0.12	-0.03	0.04	0.21	-0.26	-0.10	-0.44	phosphoribosyl-amp cyclohydrolase; phosphoribosyl-ATP pyrophosphatase [b2026]
hpf	0.11	-0.15	-0.37	-0.95	0.05	-0.78	-1.38	probable sigma-54 modulation protein [b3203]
hyaA	-0.02	0.10	-1.22	-1.59	-2.27	-2.90	-3.20	hydrogenase-1 small subunit [b0972]
hybC	-0.23	0.17	-1.34	-1.28	-1.12	-1.84	-1.97	"probable large subunit, hydrogenase-2 [b2994]"
idi	-0.13	-0.19	0.01	-0.04	0.00	-1.45	-2.27	putative enzyme [b2889]
ivy	0.05	-0.67	-1.01	-1.43	-1.22	-1.44	-1.63	"orf, hypothetical protein [b0220]"
lacZ	0.09	0.10	0.27	0.53	-0.77	-1.26	-2.03	beta-D-galactosidase [b0344]
lolB	0.17	-0.09	-0.61	-0.51	-0.57	-0.72	-1.12	"an enzyme in main pathway of synthesis of 5-aminolevulinate, possibly glutamyl-tRNA dehydrogenase [b1209]"
nadR	0.03	0.07	-0.28	-0.21	-0.48	-1.22	-1.58	probable nadAB transcriptional regulator [b4390]
nuoE	0.18	0.32	-0.75	-0.19	-0.26	-1.30	-1.06	NADH dehydrogenase I chain E [Z3544]
panC	-0.22	-0.16	-0.50	-0.49	-0.58	-0.75	-1.14	pantothenate synthetase [ECs0137]
panC	-0.22	-0.16	-0.50	-0.49	-0.58	-0.75	-1.14	pantothenate synthetase [ECs0137]
panE	0.15	0.05	-0.36	-0.17	-0.61	-0.82	-1.12	"involved in thiamin biosynthesis, alternative pyrimidine biosynthesis [b0425]"
papK	0.00	1.18	0.00	0.12	0.04	0.18	0.07	PapK protein [c_3586]
poxB	0.02	-0.28	-0.79	-1.07	-1.28	-1.25	-1.36	pyruvate oxidase [Z1105]
psiF	-0.17	-0.54	-1.50	-1.39	-0.75	-2.53	-3.44	phosphate starvation-induced protein psiF [ECs0434]
ptrB	-0.03	-0.13	-0.54	-0.37	-0.87	-1.06	-1.25	protease II [b1845]
rbsB	-0.22	-0.49	-1.11	-1.21	-1.97	-2.39	-2.94	D-ribose periplasmic binding protein [b3751]
rmf	-0.44	-0.80	-2.64	-2.68	-0.24	-2.79	-4.14	ribosome modulation factor [b0953]
rpoS	-0.14	-0.42	-0.87	-0.90	-1.48	-1.22	-1.42	"RNA polymerase, sigma S [b2741]"
sbmC	0.96	0.03	-0.76	-0.69	-1.47	-1.07	-1.86	SbmC protein [b2009]
sepL	0.34	0.16	0.12	0.04	-0.13	0.01	-0.03	sepL [Z5108]
slt	-0.02	-0.16	-0.55	-0.50	-0.73	-0.99	-1.09	soluble lytic murein transglycosylase [b4392]
speA	-0.14	-0.07	-0.42	-0.42	-0.86	-0.91	-1.23	biosynthetic arginine decarboxylase [b2938]
sufA	-0.03	-0.25	-0.74	-1.10	-1.76	-1.87	-2.26	"orf, hypothetical protein [b1684]"
sufB	0.01	-0.28	-0.82	-1.06	-1.39	-1.49	-2.00	"orf, hypothetical protein [b1683]"
tpiA	-0.55	-0.88	-1.50	-1.50	-1.48	-1.98	-1.80	triosephosphate isomerase [b3919]
treF	-0.15	-0.23	-0.46	-0.82	-0.79	-1.31	-1.44	cytoplasmic trehalase [b3519]
uspB	0.25	-0.54	-1.24	-1.22	-1.26	-2.28	-2.70	"orf, hypothetical protein [b3494]"
uspD	-0.41	-0.35	-0.85	-1.63	0.04	-2.22	-2.42	putative regulator [b3923]
uvrB	0.10	0.01	-0.53	-0.35	-0.60	-0.70	-1.05	DNA repair; excision nuclease subunit B [b0779]

wrbA	0.14	0.08	-1.97	-2.49	-2.18	-3.32	-3.56	trp repressor binding protein; affects association of trp repressor and operator [b1004]
yacK	0.72	0.75	-0.20	0.22	-0.38	-0.48	-0.71	"orf, hypothetical protein [Z0133]"
yadK	0.10	-0.05	0.50	0.07	0.80	0.90	1.09	putative fimbrial protein [Z0147]
yaeB	-0.11	-0.35	-0.44	-0.51	-0.48	-0.90	-1.10	"orf, hypothetical protein [b0195]"
yagF	-0.39	-1.27	-1.94	-1.93	-2.33	-2.66	-2.93	putative dehydratase [b0269]
yahI	0.97	0.44	0.12	0.15	-0.07	-0.01	-0.02	putative kinase [Z0412]
yajB	0.10	0.46	0.07	0.10	-0.02	-0.48	-0.51	putative glycoprotein [Z0503]
ybaY	0.45	0.00	-0.35	-0.18	-1.41	-1.35	-1.63	glycoprotein/polysaccharide metabolism [b0453]
ybbI	-0.28	-0.39	-0.46	-1.24	-2.15	-2.63	-2.68	putative transcriptional regulator [Z0636]
ybbJ	0.20	-0.10	-0.73	-0.39	-0.84	-1.51	-1.56	"orf, hypothetical protein [Z0641]"
ybbU	-0.08	-0.21	-0.79	-0.86	-1.13	-2.11	-2.28	Negative regulator of allantoin and glyoxylate utilization [c_0621]
ybdK	0.05	-0.05	-0.76	-0.79	-0.83	-1.39	-1.62	"orf, hypothetical protein [b0581]"
ybeL	-0.63	-0.07	-1.02	-1.10	-1.76	-2.05	-2.34	putative alpha helical protein [b0643]
ybgK	0.32	0.40	-0.78	-0.43	-0.41	-0.94	-0.96	putative carboxylase [b0712]
ybhL	0.18	-0.19	-1.16	-0.57	-0.81	-1.93	-2.02	"orf, hypothetical protein [b0786]"
ybhR	-0.29	-0.38	-0.09	-0.03	-0.39	-1.31	-2.03	"orf, hypothetical protein [Z1012]"
ybiM	-0.28	-0.89	-1.02	-1.35	-1.59	-2.22	-2.58	"orf, hypothetical protein [b0806]"
ybjP	-0.16	-0.26	-0.82	-1.10	-1.59	-2.39	-2.88	putative enzyme [Z1095]
ycbB	0.06	-0.64	-1.08	-1.15	-1.11	-2.14	-1.85	putative amidase [b0925]
yccJ	-0.48	-0.29	-1.70	-2.21	-1.80	-3.07	-3.19	"orf, hypothetical protein [b1003]"
yceH	-0.10	-0.42	-0.62	-0.26	-0.53	-1.22	-1.49	Hypothetical protein yceH [c_1334]
ycfH	-0.13	-0.37	-0.58	-0.79	-0.58	-1.85	-2.12	"orf, hypothetical protein [b1100]"
yddE	-0.17	-0.04	-0.37	-0.19	-0.40	-0.61	-1.13	Hypothetical protein yddE [c_1896]
ydhS	0.55	-0.22	-0.49	-0.56	-0.70	-1.42	-2.04	"orf, hypothetical protein [b1668]"
ydiH	-0.81	-0.67	-1.19	-1.98	-2.10	-2.28	-3.00	"orf, hypothetical protein [b1685]"
yebV	-0.11	-1.04	-1.42	-2.41	-2.21	-2.25	-2.61	"orf, hypothetical protein [b1836]"
yebW	0.30	-0.01	-0.09	-0.33	-0.79	-0.70	-1.22	Hypothetical protein yebW [c_2246]
yedP	-0.01	-0.38	-1.12	-0.91	-1.05	-1.65	-2.31	"orf, hypothetical protein [b1955]"
yedU	-0.07	-0.16	-0.85	-1.71	-2.18	-1.91	-2.39	Protein yedU [c_2385]
yegH	0.08	0.16	-0.30	-0.28	-0.61	-0.27	-0.75	Hypothetical protein yegH [c_2590]
yegP	-0.17	-0.68	-1.99	-2.87	-3.36	-4.22	-4.08	Hypothetical protein yegP [c_2606]
yegS	0.09	-0.10	-0.04	-0.09	0.01	-0.43	-0.32	"orf, hypothetical protein [b2086]"
yeiA	-0.23	-0.28	-1.36	-0.99	-1.87	-2.10	-2.18	putative oxidoreductase [b2147]
yejA	0.13	-0.37	-0.44	-0.69	-0.54	-0.99	-1.38	"orf, hypothetical protein [b2177]"
yfgC	-0.25	-0.30	-0.70	-0.78	-0.94	-1.12	-1.44	Hypothetical protein yfgC precursor [c_3011]
yfhM	-0.16	0.08	-0.31	-0.35	-0.77	-1.08	-1.60	"orf, hypothetical protein [b2520]"
yfiQ	-0.15	-0.64	-0.77	-0.75	-1.05	-1.42	-1.41	"orf, hypothetical protein [b2584]"

ygaT	-0.05	0.02	-0.08	-0.30	-0.34	-0.58	-1.10	Hypothetical protein ygaT [c_3207]
ygaU	-0.17	-0.16	-1.23	-2.16	-2.20	-2.75	-2.50	"orf, hypothetical protein [b2665]"
ygdR	-0.06	-0.48	-0.50	-0.66	-0.94	-1.35	-1.51	"orf, hypothetical protein [b2833]"
ygeY	-0.16	-0.14	-1.18	-1.41	-1.41	-1.50	-2.15	Hypothetical protein ygeY [c_3450]
ygfK	-0.28	-0.20	-0.94	-0.74	-1.77	-2.21	-3.00	"putative oxidoreductase, Fe-S subunit [b2878]"
ygfU	-0.18	-0.22	-0.86	-1.04	-1.56	-2.05	-2.41	putative permease [b2888]
yghZ	-0.30	-0.06	-1.19	-1.22	-1.01	-1.70	-1.96	putative reductase [b3001]
ygiC	0.19	0.15	-0.80	-0.54	-0.89	-0.94	-1.30	putative synthetase/amidase [b3038]
ygiW	-0.12	0.04	-1.05	-1.58	-1.39	-2.03	-2.08	"orf, hypothetical protein [b3024]"
yhbO	-0.11	-0.57	-0.86	-0.90	-0.98	-1.47	-1.81	"orf, hypothetical protein [b3153]"
yhgE	-0.06	-0.08	-0.11	-0.11	-0.03	-0.37	-0.39	Hypothetical protein yhgE [c_4173]
yhhA	-0.88	-0.69	-1.27	-1.36	-1.78	-2.40	-3.18	"orf, hypothetical protein [b3448]"
yifK	-0.02	-0.26	-0.50	-0.57	-1.41	-1.90	-2.62	putative amino acid/amine transport protein [b3795]
yigZ	0.13	0.06	-0.78	-0.50	-0.14	-0.45	-0.54	"orf, hypothetical protein [b3848]"
yiiS	-0.50	-0.41	-1.35	-1.81	-1.84	-2.47	-2.18	"orf, hypothetical protein [Z5467]"
yiiS	-0.50	-0.41	-1.35	-1.81	-1.84	-2.47	-2.18	"orf, hypothetical protein [Z5467]"
yiiT	-0.10	-0.57	-1.60	-1.91	-1.43	-2.45	-2.30	Hypothetical protein yiiT [c_4875]
yjbH	0.05	-0.03	0.33	0.25	0.01	0.09	-0.30	"orf, hypothetical protein [b4029]"
yjbJ	-0.15	-0.46	-1.55	-1.58	-2.06	-3.17	-3.14	"orf, hypothetical protein [b4045]"
yjbR	-0.25	0.10	-0.37	-1.37	-1.52	-1.91	-2.02	"orf, hypothetical protein [b4057]"
yjdN	-0.23	-0.37	-1.02	-1.45	-1.61	-1.99	-2.60	"orf, hypothetical protein [b4107]"
yjfY	-0.03	-0.33	-0.29	-0.62	-1.13	-1.99	-2.09	"orf, hypothetical protein [b4199]"
ypdA	0.15	0.33	0.24	-0.20	-0.26	-0.56	-0.72	putative sensor protein [b2380]
yphF	-0.02	0.57	0.10	-0.15	-0.15	-0.75	-0.90	putative LACI-type transcriptional regulator [b2548]
yqjK	0.23	0.09	-1.05	-1.33	-1.20	-1.65	-1.53	Hypothetical protein yqjK [c_3858]
yraM	0.06	0.11	0.01	-0.47	-0.58	-0.70	-0.97	putative glycosylase [b3147]
yraR	0.07	-0.43	-0.44	-0.27	-0.31	-0.56	-0.84	"orf, hypothetical protein [Z4511]"
Z1099	-0.03	-0.39	-0.65	-0.62	-0.87	-1.31	-1.31	"orf, hypothetical protein [Z1099]"
Z1769	-0.18	0.10	-0.11	-0.01	-0.24	-0.28	-0.83	unknown protein encoded by prophage CP-933N [Z1769]
Z1924	0.20	0.67	-1.09	-1.02	-1.16	-2.11	-2.74	unknown protein encoded by prophage CP-933X [Z1924]
Z2665	0.21	-0.08	-0.40	-0.26	-0.63	-1.27	-1.36	"orf, hypothetical protein [Z2665]"
Z2691	-0.09	0.34	-0.56	-0.39	-1.13	-1.56	-2.07	possible enzyme [Z2691]
Z3249	-0.10	-0.64	-1.82	-2.56	-3.16	-4.76	-3.96	"orf, hypothetical protein [Z3249]"
Z3348	1.45	1.26	0.51	0.11	-0.18	0.11	-0.09	unknown protein encoded within prophage CP-933V [Z3348]
Z3787	0.15	0.27	-0.38	-0.39	-0.70	-1.26	-1.24	"orf, hypothetical protein [Z3787]"
Z3942	0.58	0.66	0.36	0.33	-0.02	-0.05	-0.27	orf Other or unknown [Z3942]
Z4333	0.11	0.67	0.10	0.06	-0.12	0.11	-0.06	putative cytotoxin [Z4333]

Z5001	0.80	0.18	-0.01	0.04	-0.73	-1.13	-1.18	putative permease [Z5001]
Z5094	0.24	0.49	-0.06	0.07	0.14	-0.46	-0.04	unknown protein encoded within prophage CP-933L [Z5094]
Z5102	0.05	0.73	0.38	0.19	0.14	0.37	0.21	orf Unknown function [Z5102]
Z5200	0.20	1.13	0.43	0.38	-0.03	0.06	-0.02	orf Unknown function [Z5200]
Z5816	0.16	0.05	-0.29	-0.20	-0.37	-0.48	-0.72	putative virulence protein [Z5816]
Z5954	-0.01	0.67	0.00	0.14	0.25	0.01	0.03	orf Unknown function [Z5954]
