# Supplementary material for: A Man-Made ATP-Binding Protein Evolved Independent of Nature Causes Abnormal Growth in Bacterial Cells
Source: PLoS One. 2009 Oct 8;4(10):e7385. doi: 10.1371/journal.pone.0007385 (PMC2754611; doi:10.1371/journal.pone.0007385)
Supplement: Table S4 — Time dependent expression of chemotaxis genes. (0.09 MB PDF) [file pone.0007385.s006.pdf]

Gene	Hours post induction ($\log_2$(induced / un-induced))							Gene Function
	0.5	1	1.5	2	3	3.5	4	
aer	-0.02	-0.38	-0.26	-0.41	-0.19	0.34	-0.04	"aerotaxis sensor receptor, flavoprotein [b3072]"
chaC	-0.09	-0.10	-0.18	-0.48	-0.58	-0.54	-0.87	cation transport regulator [b1218]
cheA	-0.10	-0.08	0.11	-0.40	0.04	0.62	0.30	sensory transducer kinase between chemo- signal receptors and CheB and CheY [b1888]
cheB	-0.06	-0.24	-0.01	-0.14	0.22	0.25	0.36	response regulator for chemotaxis [b1883]
cheR	-0.02	-0.13	-0.02	-0.12	0.22	0.19	0.54	response regulator for chemotaxis; protein glutamate methyltransferase [b1884]
cheW	-0.20	-0.06	-0.20	0.06	-0.12	-0.23	-0.13	positive regulator of CheA protein activity [b1887]
cheY	-0.16	-0.41	-0.31	-0.38	-0.29	0.81	0.76	chemotaxis regulator transmits chemoreceptor signals to flagelllar motor components [b1882]
cheZ	-0.30	-0.02	-0.08	-0.23	0.72	0.66	0.55	Chemotaxis protein cheZ [c_2296]
csrA	-1.61	-1.10	-1.14	-1.26	-1.75	-1.27	-2.05	"carbon storage regulator; controls glycogen synthesis, gluconeogenesis, cell size and surface properties [b2696]"
dsbB	-0.88	-1.47	0.12	0.82	0.14	0.40	0.24	reoxidizes DsbA protein following formation of disulfide bond in P-ring of flagella. [b1185]
flgA	-0.08	0.20	0.07	-0.11	-0.05	0.11	0.00	flagellar biosynthesis; assembly of basal-body periplasmic P ring [b1072]
flgB	-0.05	0.04	0.07	-0.22	0.09	0.34	-0.08	"flagellar biosynthesis, cell-proximal portion of basal-body rod [b1073]"
flgC	0.01	0.04	0.07	-0.31	0.02	0.56	0.19	"flagellar biosynthesis, cell-proximal portion of basal-body rod [b1074]"
flgD	-0.07	-0.01	0.03	-0.55	0.10	0.48	0.23	"flagellar biosynthesis, initiation of hook assembly [b1075]"
flgE	-0.11	-0.16	-0.14	-0.33	0.13	0.23	0.28	"flagellar biosynthesis, hook protein [b1076]"
flgF	-0.04	0.35	0.14	-0.15	-0.40	-0.08	-0.21	"flagellar biosynthesis, cell-proximal portion of basal-body rod [b1077]"
flgG	0.14	0.20	0.12	-0.20	0.13	-0.23	0.24	"flagellar biosynthesis, cell-distal portion of basal-body rod [b1078]"
flgH	-0.02	-0.09	-0.03	-0.25	-0.12	0.60	0.30	"flagellar biosynthesis, basal-body outer-membrane L [b1079]"
flgI	-0.09	0.18	-0.15	-0.47	-0.12	0.55	0.45	homolog of Salmonella P-ring of flagella basal body [b1080]
flgJ	0.00	0.06	0.03	-0.27	-0.05	0.49	0.25	flagellar biosynthesis [b1081]
flgK	-0.12	-0.39	0.04	-0.17	0.00	-0.10	-0.21	"flagellar biosynthesis, hook-filament junction protein 1 [b1082]"
flgL	-0.18	-0.26	-0.04	-0.14	-0.16	0.01	0.23	flagellar biosynthesis; hook-filament junction protein [b1083]
flgM	-0.85	-0.11	-0.59	-0.59	-0.66	-0.65	-0.84	anti-FliA [b1071]
flgN	-0.33	-0.23	-0.87	-0.80	-0.89	-0.89	-1.35	protein of flagellar biosynthesis [b1070]
flhA	0.04	0.00	0.13	-0.05	-0.05	0.37	0.04	flagellar biosynthesis; possible export of flagellar proteins [b1879]
flhC	-0.43	-0.19	2.11	2.29	1.20	2.74	1.84	regulator of flagellar biosynthesis acting on class 2 operons; transcription initiation factor [b1891]
flhD	-0.46	-0.49	1.91	2.13	1.12	3.80	3.22	"regulator of flagellar biosynthesis, acting on class 2 operons; transcriptional initiation factor [b1892]"
fliA	0.00	0.05	0.22	0.01	0.03	0.39	0.18	flagellar biosynthesis; alternative sigma factor 28; regulation of flagellar operons [b1922]

fliC	0.03	0.00	-0.10	0.08	0.28	0.12	0.12	Flagellin [c_2338]
fliD	0.03	0.43	0.09	-0.24	-0.10	0.36	0.05	flagellar biosynthesis; filament capping protein; enables filament assembly [b1924]
fliE	-0.04	0.38	0.07	-0.17	0.43	0.35	0.46	"flagellar biosynthesis; basal-body component, possibly at [b1937]"
fliF	0.11	0.03	-0.06	-0.08	0.30	0.12	0.23	flagellar biosynthesis; basal-body MS [b1938]
fliG	0.00	0.29	-0.10	-0.15	0.10	0.46	0.06	"flagellar biosynthesis, component of motor switching and energizing, enabling rotation and determining its direction [b1939]"
fliH	0.05	0.27	-0.05	-0.28	0.06	0.22	0.09	flagellar biosynthesis; export of flagellar proteins? [b1940]
fliJ	0.00	0.00	-0.07	-0.16	-0.06	0.46	0.51	flagellar fliJ protein [b1942]
fliK	0.14	0.01	0.02	-0.13	-0.07	0.18	0.23	flagellar hook-length control protein [b1943]
fliL	-0.18	0.05	0.02	-0.26	0.05	0.44	0.44	flagellar biosynthesis [b1944]
fliM	0.00	0.54	0.07	-0.28	0.13	0.30	0.32	"flagellar biosynthesis, component of motor switch and energizing, enabling rotation and determining its direction [b1945]"
fliN	-0.11	-0.22	-0.13	-0.20	0.31	0.36	0.31	"flagellar biosynthesis, component of motor switch and energizing, enabling rotation and determining its direction [b1946]"
fliO	-0.12	-0.25	0.00	-0.34	0.16	0.60	0.61	flagelar biosynthesis [Z3037]
fliP	-0.01	-0.13	0.03	-0.19	0.28	0.56	0.51	Flagellar biosynthetic protein fliP precursor [c_2365]
fliS	-0.09	-0.05	0.02	-0.16	0.06	0.46	0.44	Flagellar protein fliS [c_2340]
fliT	1.37	1.07	0.33	0.39	0.13	-0.17	0.09	flagellar biosynthesis; repressor of class 3a and 3b operons [b1926]
malE	-0.33	0.12	-2.27	-3.35	-2.58	-3.04	-1.15	periplasmic maltose-binding protein; substrate recognition for transport and chemotaxis [b4034]
motA	-0.01	-0.17	0.65	0.92	0.56	1.03	1.03	proton conductor component of motor; no effect on switching [b1890]
motB	0.06	-0.09	0.24	0.30	0.51	0.41	0.47	"enables flagellar motor rotation, linking torque machinery to cell wall [b1889]"
mqsR	0.14	-0.61	0.54	0.39	0.16	0.42	0.66	"orf, hypothetical protein [b3022]"
nikA	-0.02	-0.13	-0.02	-0.01	-0.05	0.26	0.17	periplasmic binding protein for nickel [b3476]
tap	-0.02	0.01	0.11	-0.34	0.03	0.44	0.16	"methyl-accepting chemotaxis protein IV, peptide sensor receptor [b1885]"
tar	-0.02	0.13	-0.05	-0.39	0.31	0.06	0.04	"methyl-accepting chemotaxis protein II, aspartate sensor receptor [b1886]"
trg	0.14	0.05	-0.07	-0.04	-0.36	0.13	-0.10	"methyl-accepting chemotaxis protein III, ribose sensor receptor [b1421]"
tsr	-0.05	-0.02	-0.04	-0.02	-0.07	0.27	0.02	Methyl-accepting chemotaxis protein I [c_5430]
ycgR	-0.10	-0.06	-0.16	-0.28	0.13	0.48	0.28	"orf, hypothetical protein [b1194]"
yehP	0.00	0.20	-0.04	0.02	-0.21	0.24	-0.05	"orf, hypothetical protein [b2121]"
yeiA	-0.23	-0.28	-1.36	-0.99	-1.87	-2.10	-2.18	putative oxidoreductase [b2147]
yhjH	0.02	-0.51	0.02	-0.10	0.32	0.37	0.47	"orf, hypothetical protein [b3525]"
