# Supplementary material for: A Man-Made ATP-Binding Protein Evolved Independent of Nature Causes Abnormal Growth in Bacterial Cells
Source: PLoS One. 2009 Oct 8;4(10):e7385. doi: 10.1371/journal.pone.0007385 (PMC2754611; doi:10.1371/journal.pone.0007385)
Supplement: Table S5 — Time dependent expression of sulfur metabolism genes. (0.07 MB PDF) [file pone.0007385.s007.pdf]

Gene	Hours post induction (log_2(induced / un-induced))							Gene Function
	0.5	1	1.5	2	3	3.5	4	
aslA	0.02	-0.25	-0.17	-0.26	-0.17	-0.16	-0.37	arylsulfatase [b3801]
cbl	0.35	-0.27	1.29	0.01	0.87	0.12	0.92	transcriptional regulator cys regulon; accessory regulatory circuit affecting cysM [b1987]
csdA	-0.02	-0.09	0.07	0.23	0.00	0.47	0.15	"orf, hypothetical protein [b2810]"
csdE	0.09	-0.12	-0.07	0.02	0.10	-0.27	-0.87	"orf, hypothetical protein [b2811]"
cysA	-0.09	-0.42	-0.12	-0.17	-0.15	0.39	-0.02	Sulfate transport ATP-binding protein cysA [c_2956]
cysC	-0.12	-0.59	0.06	-0.12	0.03	0.05	0.11	adenosine 5-phosphosulfate kinase [b2750]
cysD	0.08	-0.04	-0.26	-0.32	-0.08	-0.40	0.11	ATP:sulfurylase [b2752]
cysH	0.01	-0.21	-0.16	-0.04	0.04	0.19	0.15	3-phosphoadenosine 5-phosphosulfate reductase [b2762]
cysI	0.07	-0.16	-0.05	-0.11	0.09	0.19	0.22	"sulfite reductase, alpha subunit [b2763]"
cysJ	0.16	-0.22	-0.17	-0.05	0.39	-0.33	0.14	Sulfite reductase [c_3323]
cysN	-0.04	-0.24	-0.18	-0.08	-0.06	0.14	0.15	ATP-sulfurylase [b2751]
cysP	-0.04	-0.03	-0.15	-0.31	0.05	0.08	-0.09	thiosulfate binding protein [Z3690]
cysQ	-0.05	-0.37	-0.65	-0.82	-0.72	-0.75	-1.23	affects pool of 3-phosphoadenosine-5-phosphosulfate in pathway of sulfite synthesis [b4214]
cysU	-0.18	-0.16	0.95	-0.21	0.94	0.38	0.43	Sulfate transport system permease protein cysT [c_2958]
cysW	0.00	0.20	-0.06	-0.06	-0.05	0.43	0.31	sulfate transport system permease W protein [b2423]
cysZ	0.07	-0.38	0.40	0.42	0.27	0.12	0.39	required for sulfate transport [b2413]
dcyD	0.49	0.32	-0.89	-0.80	-0.99	-0.66	-0.13	putative 1-aminocyclopropane-1-carboxylate deaminase [b1919]
dipZ	0.15	-0.69	-1.48	-1.22	-0.60	-0.35	-0.21	thiol:disulfide interchange protein; copper tolerance [b4136]
iscU	-0.31	0.29	0.65	0.92	1.07	2.26	2.21	"orf, hypothetical protein [b2529]"
sbp	-0.38	0.22	0.33	0.02	-0.11	0.22	0.50	periplasmic sulfate-binding protein [b3917]
sseA	-0.65	-0.74	-0.81	-0.55	-0.97	-1.15	-1.72	putative thiosulfate sulfurtransferase [b2521]
sseB	-0.23	-0.17	1.57	1.65	1.17	0.98	1.43	enhanced serine sensitivity [b2522]
ssuB	-0.04	-0.21	-0.17	-0.24	-0.01	0.19	0.09	putative ATP-binding component of a transport system [b0933]
ssuD	0.01	-0.37	0.08	-0.32	0.17	0.43	0.34	"orf, hypothetical protein [b0935]"
ssuE	-0.10	-0.37	0.13	-0.29	0.39	0.42	0.61	"orf, hypothetical protein [b0937]"
sufA	-0.03	-0.25	-0.74	-1.10	-1.76	-1.87	-2.26	"orf, hypothetical protein [b1684]"
sufB	0.01	-0.28	-0.82	-1.06	-1.39	-1.49	-2.00	"orf, hypothetical protein [b1683]"
sufC	0.12	-0.10	-1.26	-1.25	-1.23	-1.45	-1.85	putative ATP-binding component of a transport system [b1682]
sufD	0.05	0.35	-1.02	-1.21	-1.61	-1.47	-1.89	"orf, hypothetical protein [b1681]"
sufE	0.00	0.00	-0.07	-0.02	0.00	0.00	-0.43	"orf, hypothetical protein [b1679]"
sufS	-0.24	-0.20	-0.66	-0.92	-1.15	-0.51	-0.92	"orf, hypothetical protein [b1680]"
tauA	-0.18	0.64	0.16	-0.20	0.20	0.36	0.41	Taurine-binding periplasmic protein precursor [c_0472]
tauB	0.05	0.01	0.07	0.06	-0.04	0.76	0.55	taurine ATP-binding component of a transport system [b0366]

tauC	-0.04	-0.20	0.18	-0.07	0.36	0.38	0.60	taurine transport system permease protein [b0367]
tauD	-0.03	-0.20	0.08	0.01	0.04	0.42	0.38	"taurine dioxygenase, 2-oxoglutarate-dependent [Z0467]"
ydeN	0.00	-0.35	-2.86	-3.09	-0.67	-0.46	-0.23	putative sulfatase [b1498]
yidJ	-0.02	-0.33	0.02	-0.35	0.19	0.01	0.28	Putative sulfatase yidJ [c_4601]
yjcS	0.02	-0.09	0.45	0.02	0.54	0.26	0.52	"orf, hypothetical protein [b4083]"
ynjE	-0.07	-0.36	-0.50	-0.28	-0.19	-1.14	-0.86	putative thiosulfate sulfur transferase [b1757]
