# Supplementary material for: A Man-Made ATP-Binding Protein Evolved Independent of Nature Causes Abnormal Growth in Bacterial Cells
Source: PLoS One. 2009 Oct 8;4(10):e7385. doi: 10.1371/journal.pone.0007385 (PMC2754611; doi:10.1371/journal.pone.0007385)
Supplement: Table S6 — Time dependent expression of the rpoS regulon. (0.08 MB PDF) [file pone.0007385.s008.pdf]

Gene	Hours post induction ($\log_2$(induced / un-induced))							Gene Function
	0.5	1	1.5	2	3	3.5	4	
aidB	0.21	-0.11	-0.80	-1.19	-1.00	-1.82	-2.00	putative acyl coenzyme A dehydrogenase [b4187]
aldB	-0.06	-0.31	-1.42	-1.34	-1.65	-2.39	-1.91	aldehyde dehydrogenase B [b3588]
appB	0.03	-0.38	-0.65	-1.71	-1.52	-1.36	-1.34	"probable third cytochrome oxidase, subunit II [b0979]"
argH	-0.28	-0.26	-0.28	0.00	-0.02	-0.21	-0.51	argininosuccinate lyase [b3960]
aroM	-0.07	-0.17	1.29	1.40	1.40	1.76	1.66	AroM protein [c_0498]
dps	-0.13	-0.87	-2.02	-1.87	-1.99	-1.03	-2.36	"global regulator, starvation conditions [b0812]"
gabD	0.42	0.39	2.01	0.54	1.24	0.32	0.82	"succinate-semialdehyde dehydrogenase, NADP-dependent activity [b2661]"
gabP	-0.23	-0.08	-0.34	-0.48	-0.52	-0.65	-0.80	transport permease protein of gamma-aminobutyrate [b2663]
hdeB	-0.56	-1.47	-2.04	-1.43	-1.29	-1.41	-1.63	"orf, hypothetical protein [b3509]"
katE	-0.01	0.19	-1.50	-1.70	-1.78	-1.56	-1.44	catalase; hydroperoxidase HPII [b1732]
katG	-0.18	-0.43	-0.97	-0.83	-0.88	-1.98	-1.19	catalase; hydroperoxidase HPI [b3942]
mltB	0.07	-0.37	0.07	0.22	-0.06	-0.07	-0.59	membrane-bound lytic murein transglycosylase B [b2701]
narY	0.22	0.35	-0.33	-0.26	-0.59	-0.17	-0.43	Respiratory nitrate reductase 2 beta chain [c_1899]
osmY	0.02	0.50	0.51	-0.05	-1.30	-1.18	-1.83	hyperosmotically inducible periplasmic protein [b4376]
otsA	0.28	0.78	0.02	-0.72	-0.39	-1.10	-1.33	trehalose-6-phosphate synthase [b1896]
otsA	0.28	0.78	0.02	-0.72	-0.39	-1.10	-1.33	trehalose-6-phosphate synthase [b1896]
phnP	-0.22	-0.21	-0.45	-0.21	-0.25	-0.11	-0.33	PhnP protein [c_5098]
poxB	0.02	-0.28	-0.79	-1.07	-1.28	-1.25	-1.36	pyruvate oxidase [Z1105]
rpoS	-0.14	-0.42	-0.87	-0.90	-1.48	-1.22	-1.42	"RNA polymerase, sigma S [b2741]"
talA	-0.20	0.08	-1.04	-1.79	-2.20	-1.58	-1.82	transaldolase A [b2464]
ugpC	0.04	0.49	-0.04	-0.40	-0.16	-0.21	-0.58	ATP-binding component of sn-glycerol 3-phosphate transport system [b3450]
ugpE	0.01	-0.19	0.00	-0.27	0.00	-0.28	-0.82	"sn-glycerol 3-phosphate transport system, integral membrane protein [b3451]"
uspB	0.25	-0.54	-1.24	-1.22	-1.26	-2.28	-2.70	"orf, hypothetical protein [b3494]"
wrbA	0.14	0.08	-1.97	-2.49	-2.18	-3.32	-3.56	trp repressor binding protein; affects association of trp repressor and operator [b1004]
ybaY	0.45	0.00	-0.35	-0.18	-1.41	-1.35	-1.63	glycoprotein/polysaccharide metabolism [b0453]
ybiO	-0.08	-0.18	-0.28	-0.32	-0.35	-1.13	-1.06	putative transport protein [b0808]
ydaM	-0.39	0.12	0.04	-0.15	-0.76	-0.54	-0.76	"orf, hypothetical protein [b1341]"
ydcS	-0.06	-0.32	-1.03	-0.64	-0.87	-1.67	-1.33	Putative ABC transporter Periplasmic binding protein ydcS [c_1864]
yeaG	-0.10	-0.22	-1.79	-1.32	-1.27	-2.41	-2.58	"orf, hypothetical protein [b1783]"
yebF	0.27	-0.09	0.51	0.51	-0.09	0.33	0.62	"orf, hypothetical protein [b1847]"
yehX	0.15	-0.61	-1.01	-0.87	-0.56	-0.82	-0.45	putative ATP-binding component of a transport system [b2129]
yfcG	-0.04	-0.61	-0.74	-0.50	-0.67	-1.72	-1.52	putative S-transferase [b2302]
ygaF	-0.01	-0.12	-0.54	-0.48	-0.38	-1.03	-1.08	Hypothetical protein ygaF [c_3208]
ygaU	-0.17	-0.16	-1.23	-2.16	-2.20	-2.75	-2.50	"orf, hypothetical protein [b2665]"
ygdI	-0.35	-1.06	-1.03	-0.99	-1.45	-1.99	-2.05	"orf, hypothetical protein [b2809]"

yhiN	0.39	0.09	0.87	0.43	0.74	-0.24	0.16	"orf, hypothetical protein [b3492]"
yhjD	-0.01	-0.41	-1.09	-1.51	-1.17	-1.72	-1.66	"orf, hypothetical protein [b3522]"
yhjG	0.00	-0.19	-0.65	-0.61	-0.59	-1.64	-1.41	"orf, hypothetical protein [b3524]"
yhjY	-0.18	-0.14	-0.13	-0.42	-0.67	-0.61	-0.73	Hypothetical protein yhjY [c_4366]
yjbE	-0.45	-0.73	-0.63	-0.59	-0.80	-0.01	-0.28	"orf, hypothetical protein [b4026]"
yjbJ	-0.15	-0.46	-1.55	-1.58	-2.06	-3.17	-3.14	"orf, hypothetical protein [b4045]"
yjgR	0.04	-0.26	-0.27	-0.01	-0.28	-0.56	-0.33	"orf, hypothetical protein [b4263]"
yjiN	-0.11	0.27	0.15	0.15	0.01	0.13	0.04	Hypothetical protein yjiN [c_5419]
yliI	-0.03	-0.16	-0.15	-0.14	-0.51	-0.48	-0.67	putative dehydrogenase [b0837]
yodC	0.44	0.62	-0.98	-1.25	-1.68	-1.16	-0.91	"orf, hypothetical protein [b1957]"
yphA	-0.01	-0.83	-1.14	-1.18	-1.62	-1.55	-1.25	Hypothetical protein yphA [c_3065]
