# Supplementary material for: A Man-Made ATP-Binding Protein Evolved Independent of Nature Causes Abnormal Growth in Bacterial Cells
Source: PLoS One. 2009 Oct 8;4(10):e7385. doi: 10.1371/journal.pone.0007385 (PMC2754611; doi:10.1371/journal.pone.0007385)
Supplement: Table S7 — Time dependent expression of SOS pathway genes. (0.05 MB PDF) [file pone.0007385.s009.pdf]

Gene	Hours post induction ($\log_2$(induced / un-induced))							Gene Function
	0.5	1	1.5	2	3	3.5	4	
cho	0.02	-0.43	-0.19	-0.39	-0.06	-0.75	0.02	putative excinuclease subunit [b1741]
dinB	-0.19	-0.40	0.00	0.06	0.27	-0.23	-0.25	damage-inducible protein P; putative tRNA synthetase [b0231]
dinF	0.26	-0.17	-0.52	-0.64	0.08	0.38	0.72	DNA-damage-inducible protein F [b4044]
dinG	0.27	0.17	-1.02	-0.44	-0.68	-0.34	-0.79	probably ATP-dependent helicase [b0799]
dinI	-0.13	-0.34	-0.42	-0.14	0.13	0.50	0.14	damage-inducible protein I [b1061]
ftsK	-0.06	-0.04	-0.40	-0.16	-0.48	-0.19	0.15	cell division protein [b0890]
lexA	-0.01	-0.23	-0.09	-0.07	0.00	-0.01	-0.34	regulator for SOS [b4043]
polB	0.00	0.03	0.06	-0.07	0.22	-0.26	0.01	DNA polymerase II [b0060]
recA	-0.07	0.45	0.48	0.24	0.27	0.55	0.45	"DNA strand exchange and renaturation, DNA-dependent ATPase, DNA- and ATP-dependent coprotease [b2699]"
recN	-0.39	0.01	0.50	0.11	0.22	0.53	0.25	protein used in recombination and DNA repair [b2616]
recX	0.28	0.06	-0.01	0.33	0.49	0.17	0.37	"regulator, OraA protein [Z4001]"
rfaG	0.29	0.08	0.37	0.35	0.19	0.32	0.54	Lipopolysaccharide core biosynthesis protein rfaG [c_4455]
ruvA	0.06	-0.45	-0.24	-0.34	0.03	0.11	-0.02	Holliday junction helicase subunit B; branch migration; repair [b1861]
ruvB	0.30	0.18	-0.45	0.45	0.65	0.17	0.25	Holliday junction helicase subunit A; branch migration; repair [b1860]
sbmC	0.96	0.03	-0.76	-0.69	-1.47	-1.07	-1.86	SbmC protein [b2009]
ssb	-0.47	0.34	0.01	-0.18	-0.32	-0.11	-0.03	ssDNA-binding protein [b4059]
sulA	0.16	0.10	-0.11	-0.03	0.70	-1.14	-1.27	suppressor of lon; inhibits cell division and ftsZ ring formation [b0958]
symE	1.57	-0.08	0.22	0.51	2.02	0.21	1.77	"orf, hypothetical protein [b4347]"
umuC	0.08	1.43	-0.26	0.18	-0.06	-0.27	-0.42	SOS mutagenesis and repair [b1184]
umuD	0.23	-0.20	0.20	0.93	-0.26	0.57	0.29	SOS mutagenesis; error-prone repair; processed to UmuD; forms complex with UmuC [b1183]
uvrA	-0.18	0.46	-0.60	-0.46	-0.95	-0.70	-0.51	excision nuclease subunit A [b4058]
uvrB	0.10	0.01	-0.53	-0.35	-0.60	-0.70	-1.05	DNA repair; excision nuclease subunit B [b0779]
uvrD	0.06	0.77	0.21	0.40	1.27	0.28	1.17	DNA-dependent ATPase I and helicase II [b3813]
ybfE	-0.22	-0.15	1.56	1.40	1.57	1.71	1.99	"orf, hypothetical protein [b0685]"
ydjM	0.01	-0.22	0.25	0.76	0.09	0.51	0.38	"orf, hypothetical protein [b1728]"
yebG	-0.17	-0.71	0.03	0.48	0.66	-0.06	0.10	"orf, hypothetical protein [b1848]"
