# Supplementary material for: A Man-Made ATP-Binding Protein Evolved Independent of Nature Causes Abnormal Growth in Bacterial Cells
Source: PLoS One. 2009 Oct 8;4(10):e7385. doi: 10.1371/journal.pone.0007385 (PMC2754611; doi:10.1371/journal.pone.0007385)
Supplement: Table S8 — Time dependent expression of cell division genes. (0.10 MB PDF) [file pone.0007385.s010.pdf]

Gene	Hours post induction ($\log_2$(induced / un-induced))							Gene Function
	0.5	1	1.5	2	3	3.5	4	
amiC	-0.23	-0.09	0.68	0.68	1.28	1.62	1.52	putative amidase [b2817]
bolA	-0.24	-1.12	-1.09	-1.32	-1.46	-1.81	-2.41	possible regulator of murein genes [b0435]
cedA	0.32	-0.53	1.28	0.88	0.93	0.84	1.42	"orf, hypothetical protein [b1731]"
crcB	-0.14	-0.69	-0.50	-0.83	-0.64	0.03	-0.49	"orf, hypothetical protein [b0624]"
dacA	-0.60	-0.11	-0.15	-0.24	0.13	1.34	1.27	"D-alanyl-D-alanine carboxypeptidase, fraction A; penicillin-binding protein 5 [b0632]"
dacC	-0.30	-0.37	-0.28	-0.18	-1.02	-1.40	-0.93	D-alanyl-D-alanine carboxypeptidase; penicillin-binding protein 6 [b0839]
damX	0.12	-0.12	-0.27	-0.37	-0.79	-0.63	-0.73	putative membrane protein; interferes with cell division [b3388]
ddlB	-0.02	-0.11	-0.38	-0.07	-0.01	-0.29	-0.12	"D-alanine-D-alanine ligase B, affects cell division [b0092]"
dnaC	0.05	-0.36	-0.44	-0.46	-0.48	-0.37	-0.31	chromosome replication; initiation and chain elongation [b4361]
dnaK	-0.70	0.19	1.81	2.44	1.09	0.07	-0.26	chaperone Hsp70; DNA biosynthesis; autoregulated heat shock proteins [b0014]
envC	-0.30	-0.81	0.27	0.30	0.27	0.62	0.84	putative membrane protein [b3613]
fic	0.03	0.29	-0.90	-0.72	-0.59	-1.87	-1.76	"induced in stationary phase, recognized by rpoS, affects cell division [b3361]"
ftsA	-0.40	-0.26	-0.28	-0.27	-0.94	0.07	-0.28	"ATP-binding cell division protein, septation process, complexes with FtsZ, associated with junctions of inner and outer membranes [b0094]"
ftsB	0.05	-0.14	0.20	-0.06	0.35	-0.50	-0.14	"orf, hypothetical protein [b2748]"
ftsE	-0.04	-0.69	0.30	0.53	0.34	0.15	0.30	ATP-binding component of a membrane-associated complex involved in cell division [b3463]
ftsI	-0.09	-0.73	0.08	-0.28	-0.44	-0.59	-0.90	septum formation; penicillin-binding protein 3; peptidoglycan synthetase [b0084]
ftsK	-0.06	-0.04	-0.40	-0.16	-0.48	-0.19	0.15	cell division protein [b0890]
ftsL	0.05	-0.27	-0.15	0.12	0.00	0.24	-0.78	cell division protein; ingrowth of wall at septum [b0083]
ftsN	-0.60	-0.17	0.76	0.45	0.13	0.25	-0.42	essential cell division protein [b3933]
ftsP	0.14	-0.23	-0.46	-0.12	-0.34	-0.24	0.07	suppressor of ftsI [b3017]
ftsQ	-0.41	-0.25	-0.15	0.57	0.15	0.07	-0.16	cell division protein; ingrowth of wall at septum [b0093]
ftsW	0.39	-0.68	-0.70	-0.28	-0.23	-0.36	-0.20	cell division; membrane protein involved in shape determination [b0089]
ftsX	-0.26	-0.15	0.17	0.18	-0.15	-0.25	-0.62	cell division membrane protein [b3462]
ftsY	-0.22	0.08	0.32	0.74	-0.17	0.92	0.39	cell division membrane protein [b3464]
ftsZ	0.16	0.11	-0.42	-0.11	-0.54	-0.24	0.06	cell division; forms circumferential ring; tubulin-like GTP-binding protein and GTPase [b0095]
groL	-0.37	-0.03	0.82	0.99	0.87	-0.44	-0.05	"GroEL, chaperone Hsp60, peptide-dependent ATPase, heat shock protein [b4143]"
groS	-0.80	-0.58	0.97	1.24	0.99	0.02	0.35	"GroES, 10 Kd chaperone binds to Hsp60 in pres. Mg-ATP, suppressing its ATPase activity [b4142]"
grpE	-1.10	-1.17	0.22	0.90	0.51	0.07	0.44	phage lambda replication; host DNA synthesis; heat shock protein; protein repair [b2614]
hflB	-0.38	0.21	-0.12	0.21	-0.19	0.26	0.11	"degrades sigma32, integral membrane peptidase, cell division protein [b3178]"

hslU	-0.06	0.24	0.28	1.24	0.41	-0.30	-0.25	"heat shock protein hslVU, ATPase subunit, homologous to chaperones [b3931]"
hslV	-0.17	0.19	-0.05	1.06	0.77	0.07	0.39	"heat shock protein hslVU, proteasome-related peptidase subunit [b3932]"
lon	-0.05	0.10	0.97	1.66	1.57	0.50	0.73	"DNA-binding, ATP-dependent protease La; heat shock K-protein [b0439]"
minC	-0.42	-0.39	-0.15	0.29	-0.16	1.05	0.43	"cell division inhibitor, inhibits ftsZ ring formation [b1176]"
minD	-0.92	-0.17	-0.46	-0.53	-0.89	0.28	-0.59	"cell division inhibitor, a membrane ATPase, activates minC [b1175]"
minE	0.38	0.11	-0.40	-0.54	-0.46	1.13	0.95	"cell division topological specificity factor, reverses MinC inhibition of ftsZ ring formation [b1174]"
mltA	-0.15	-0.54	0.58	0.73	0.15	0.69	0.45	membrane-bound lytic murein transglycosylase A [b2813]
mltB	0.07	-0.37	0.07	0.22	-0.06	-0.07	-0.59	membrane-bound lytic murein transglycosylase B [b2701]
mrcA	0.14	-0.19	0.05	-0.16	-0.06	0.43	0.26	peptidoglycan synthetase; penicillin-binding protein 1A [b3396]
mrcB	1.03	0.52	0.86	0.36	1.06	0.53	0.46	peptidoglycan synthetase; penicillin-binding protein 1B [b0149]
mrdA	0.03	-0.53	0.16	0.11	-0.26	0.62	0.14	"cell elongation, e phase; peptidoglycan synthetase; penicillin-binding protein 2 [b0635]"
mrdB	-0.05	-0.01	-0.17	-0.63	-0.09	0.61	0.81	rod shape-determining membrane protein; sensitivity to radiation and drugs [b0634]
mreB	-0.19	-0.06	0.31	0.36	-0.19	0.97	0.69	"regulator of ftsI, penicillin binding protein 3, septation function [b3251]"
mukB	0.09	-0.24	-0.08	0.07	-0.07	-0.21	-0.17	kinesin-like cell division protein involved in chromosome partitioning [b0924]
mukE	0.08	0.25	0.03	0.45	0.68	0.37	0.24	"orf, hypothetical protein [b0923]"
mukF	0.03	0.22	0.14	0.67	0.15	0.47	0.29	mukF protein [Z1269]
murF	0.27	-0.16	-0.64	-0.54	-0.62	-0.50	-0.24	D-alanine:D-alanine-adding enzyme [b0086]
nlpI	-0.28	-0.62	0.03	-0.35	-0.28	0.36	-0.23	putative control proteins [b3163]
obgE	0.15	0.24	0.61	0.69	0.69	0.05	0.05	putative GTP-binding factor [b3183]
pbpG	-0.25	-0.38	0.05	-0.42	-0.48	-0.51	-1.17	penicillin-binding protein 7 [b2134]
prlC	0.19	-0.19	-0.09	0.22	-0.21	-0.52	-0.32	oligopeptidase A [Z4898]
rng	-0.02	-0.18	-0.03	-0.20	-0.47	-0.51	-0.65	bundles of cytoplasmic filaments [b3247]
rrmJ	-0.60	-0.06	-0.07	0.18	0.01	0.23	0.66	cell division protein [b3179]
sdiA	0.34	0.87	0.81	0.78	1.24	1.49	1.25	Regulatory protein sdiA [c_2330]
slmA	-0.23	-0.37	0.38	0.25	-0.68	0.52	0.13	putative transcriptional regulator [b3641]
slt	-0.02	-0.16	-0.55	-0.50	-0.73	-0.99	-1.09	soluble lytic murein transglycosylase [b4392]
sohA	-0.67	-1.03	-0.41	-0.19	-0.48	-0.02	-0.46	putative protease; htrA suppressor protein [b3129]
sulA	0.16	0.10	-0.11	-0.03	0.70	-1.14	-1.27	suppressor of lon; inhibits cell division and ftsZ ring formation [b0958]
tig	0.12	-0.02	1.19	2.41	1.39	1.28	1.72	trigger factor; a molecular chaperone involved in cell division [b0436]
tolC	-0.28	0.39	0.39	-0.04	-0.60	-0.36	-0.14	outer membrane channel; specific tolerance to colicin E1; segregation of daughter chromosomes [b3035]
xerC	-0.14	-0.16	-0.90	-0.65	-0.01	0.22	0.21	"site-specific recombinase, acts on cer sequence of [Z5328]"
yhjQ	0.39	0.16	0.33	0.37	0.49	0.60	0.52	"orf, hypothetical protein [b3534]"
yihA	-0.73	0.09	0.06	-0.19	0.18	0.99	0.60	Probable GTP-binding protein engB [c_4812]
ymgF	0.12	-0.39	0.43	0.39	1.42	0.91	2.11	predicted protein [b4520]

zapA	-0.08	-0.53	-0.95	-0.60	-0.19	-1.11	-1.75	"orf, hypothetical protein [b2910]"
zipA	-0.79	-0.40	-0.84	-0.87	-0.98	-0.48	-0.79	cell division protein involved in FtsZ ring [b2412]
