# Supplementary material for: A Man-Made ATP-Binding Protein Evolved Independent of Nature Causes Abnormal Growth in Bacterial Cells
Source: PLoS One. 2009 Oct 8;4(10):e7385. doi: 10.1371/journal.pone.0007385 (PMC2754611; doi:10.1371/journal.pone.0007385)
Supplement: Table S9 — Expression over time of genes involved in ATP biosynthesis. (0.10 MB PDF) [file pone.0007385.s011.pdf]

Gene	Hours post induction ($\log_2$(induced / un-induced))							Gene Function
	0.5	1	1.5	2	3	3.5	4	
add	-0.54	-0.58	-0.70	-0.48	-0.86	0.10	-0.14	adenosine deaminase [b1623]
adk	-0.14	-0.07	-0.02	-0.51	-0.48	1.17	0.86	adenylate kinase activity; pleiotropic effects on glycerol-3-phosphate acyltransferase activity [b0474]
amn	0.37	0.34	-0.79	-0.72	-0.42	0.12	0.17	AMP nucleosidase [b1982]
apt	-0.28	0.04	0.87	0.31	0.33	1.00	0.44	adenine phosphoribosyltransferase [b0469]
crp	-0.50	-0.45	-0.58	-0.98	-0.38	-0.34	-0.93	cyclic AMP receptor protein [b3357]
cycA	0.04	0.24	-0.06	0.34	-0.09	0.83	0.37	"transport of D-alanine, D-serine, and glycine [b4208]"
cytR	-0.44	-0.60	0.95	1.21	0.37	0.58	-0.07	"regulator for deo operon, udp, cdd, tsx, nupC, and nupG [b3934]"
deoB	-0.03	0.17	-0.63	-0.79	-1.14	-1.43	-1.33	phosphopentomutase [b4383]
deoD	-0.74	0.59	0.30	-0.58	-0.72	-0.73	-1.04	purine-nucleoside phosphorylase [b4384]
deoR	0.04	-0.15	-0.23	-0.11	-0.38	-0.11	-0.35	Deoxyribose operon repressor [c_0925]
fis	-0.50	-0.35	0.76	0.24	-0.31	0.52	0.21	site-specific DNA inversion stimulation factor; DNA-binding protein; a trans activator for transcription [b3261]
gmk	-0.44	-0.56	-0.18	-0.42	-0.19	0.27	0.15	guanylate kinase [b3648]
gmk	-0.44	-0.56	-0.18	-0.42	-0.19	0.27	0.15	guanylate kinase [b3648]
gpt	-0.26	0.33	1.82	0.85	0.50	0.52	0.77	Xanthine-guanine phosphoribosyltransferase [c_0384]
gpt	-0.26	0.33	1.82	0.85	0.50	0.52	0.77	Xanthine-guanine phosphoribosyltransferase [c_0384]
guaA	-0.15	0.28	-0.45	0.01	0.14	0.36	0.41	GMP synthetase [b2507]
guaB	-0.05	0.04	-0.12	-0.07	-0.24	0.20	0.49	Inosine-5-monophosphate dehydrogenase [c_3027]
guaC	0.21	0.29	0.33	0.24	0.64	0.92	0.75	GMP reductase [b0104]
hpt	-0.47	0.21	0.02	-0.66	-0.43	0.54	0.65	hypoxanthine phosphoribosyltransferase [b0125]
modE	0.09	-0.04	-0.51	-0.57	-0.15	0.05	-0.20	molybdate uptake regulatory protein [b0761]
ndk	0.23	-0.04	1.68	1.94	0.25	1.60	2.17	nucleoside diphosphate kinase [b2518]
phoP	-0.04	0.82	-0.04	-0.04	-0.40	-0.43	-0.12	transcriptional regulatory protein [b1130]
phoP	-0.04	0.82	-0.04	-0.04	-0.40	-0.43	-0.12	transcriptional regulatory protein [b1130]
prs	-0.26	-0.33	0.49	0.30	-0.61	0.04	-0.80	phosphoribosylpyrophosphate synthetase [b1207]
purA	-0.25	-0.12	-0.77	-0.53	-1.07	-1.06	-1.23	adenylosuccinate synthetase [b4177]
purB	-0.17	0.34	0.67	0.04	0.04	-0.02	0.22	adenylosuccinate lyase [b1131]
purC	-0.47	-0.29	-0.47	-0.51	-0.06	0.31	0.94	phosphoribosylaminoimidazole-succinocarboxamide synthetase = SAICAR synthetase [b2476]
purD	-0.20	-0.16	-0.01	-0.10	-0.49	-0.18	-0.28	phosphoribosylglycinamide synthetase = GAR synthetase [b4005]
purE	0.11	-0.03	0.04	-0.12	0.10	0.17	0.08	"phosphoribosylaminoimidazole carboxylase = AIR carboxylase, catalytic subunit [b0523]"
purF	-0.06	-0.27	0.10	0.47	-0.07	-0.69	-1.00	amidophosphoribosyltransferase = PRPP amidotransferase [Z3574]
purH	-0.12	-0.43	-0.24	-0.19	-0.05	0.12	0.12	Purine biosynthesis protein PurH [c_4964]

purK	0.11	-0.18	-0.11	-0.23	-0.01	0.21	-0.05	"phosphoribosylaminoimidazole carboxylase = AIR carboxylase, CO [b0522]"
purL	0.02	-0.37	0.18	-0.04	-0.01	0.10	0.09	phosphoribosylformyl-glycineamide synthetase = FGAM synthetase [b2557]
purM	-0.35	-0.34	0.20	0.06	-0.33	0.06	-0.10	phosphoribosylaminoimidazole synthetase = AIR synthetase [b2499]
purN	-0.14	-0.40	-0.15	0.04	-0.26	0.17	-0.70	Phosphoribosylglycinamide formyltransferase [c_3018]
purR	-0.03	-0.26	1.19	1.38	1.15	1.30	1.35	"transcriptional repressor for pur regulon, glyA, glnB, prsA, speA [b1658]"
purT	-0.08	-0.22	-0.38	-0.21	0.41	-0.10	0.09	phosphoribosylglycinamide formyltransferase 2 [b1849]
purU	0.24	-0.06	-0.12	-0.33	0.06	1.37	1.10	formyltetrahydrofolate deformylase; for purT-dependent FGAR synthesis [b1232]
			**ATP synthase genes**					
atpA	-1.33	-0.65	-0.10	0.06	-0.30	0.09	0.43	"membrane-bound ATP synthase, F1 sector, alpha-subunit [b3734]"
atpB	-1.13	-0.79	0.12	0.52	0.32	1.52	1.42	"membrane-bound ATP synthase, F0 sector, subunit a [b3738]"
atpC	0.00	-0.14	-0.67	-0.93	-0.58	-1.38	-0.67	"membrane-bound ATP synthase, F1 sector, epsilon-subunit [b3731]"
atpD	-0.15	0.30	-1.14	-0.89	-0.45	-1.39	-0.36	"membrane-bound ATP synthase, F1 sector, beta-subunit [b3732]"
atpE	-0.07	-0.56	-0.45	-0.38	-0.43	0.26	0.54	"membrane-bound ATP synthase, F0 sector, subunit c [b3737]"
atpF	-0.34	-0.04	-0.02	0.25	0.27	0.14	0.43	"membrane-bound ATP synthase, F0 sector, subunit b [b3736]"
atpG	-0.30	0.34	-0.54	-0.77	-1.07	-1.08	-0.65	"membrane-bound ATP synthase, F1 sector, gamma-subunit [b3733]"
atpH	-0.40	-0.57	-0.30	-0.08	-0.15	0.23	0.51	"membrane-bound ATP synthase, F1 sector, delta-subunit [b3735]"
atpI	-0.45	-1.08	0.96	-0.34	0.42	0.37	2.24	"membrane-bound ATP synthase, dispensable protein, affects expression of atpB [b3739]"
zntA	0.18	-0.48	-1.17	-0.13	-0.37	-0.16	-0.41	zinc-transporting ATPase [b3469]
mgtA	0.18	-0.28	-0.58	-0.46	-0.30	0.25	0.26	"Mg2+ transport ATPase, P-type 1 [b4242]"
fliI	0.77	-0.02	1.04	0.48	1.09	0.01	1.27	flagellum-specific ATP synthase [b1941]
Rho	-0.22	0.69	0.07	0.06	-0.49	0.98	0.61	transcription termination factor Rho; polarity suppressor [b3783]
